# Bacterial and Fungal Midgut Communities of Four Australian Citrus Stinkbug Species, With a Focus on *Pantoea*‐Like Symbionts and Their Fitness Effects in *Biprorulus bibax*

**DOI:** 10.1111/1462-2920.70459

**Published:** 2026-10-26

**Authors:** Alihan Katlav, Juntao Wang, Piotr Trębicki, Amir H. Tourani, Jennifer L. Morrow, Hongwei Liu, Markus Riegler

**Affiliations:** ^1^ Hawkesbury Institute for the Environment, Western Sydney University Penrith New South Wales Australia; ^2^ School of Science, Western Sydney University Penrith New South Wales Australia; ^3^ UWA School of Agriculture and Environment, University of Western Australia Perth Western Australia Australia; ^4^ Applied BioSciences, Macquarie University Sydney New South Wales Australia; ^5^ College of Agro‐Grassland Science, Nanjing Agricultural University Nanjing China

**Keywords:** bronze orange bug, gammaproteobacteria, gumtree shield bug, gut microbiota, lychee stink bug, spined citrus bug

## Abstract

True bugs harbour diverse microbial symbionts and are models of host–microbe interactions. Yet, most studies focus on bacterial partners of Holarctic‐Oriental species, leaving bacterial and fungal communities of Australasian taxa unexplored. We conducted the first comparative analysis of bacterial and fungal microbiota inhabiting the crypt‐bearing midgut region of Australian stink bugs by focussing on citrus‐associated *Biprorulus bibax* and *Poecilometis strigatus* (Pentatomidae), and *Lyramorpha rosea* and *Musgraveia sulciventris* (Tessaratomidae). Using high‐throughput amplicon sequencing, we found consistently low‐diversity bacterial communities dominated by host‐specific gammaproteobacterial symbionts within the *Pantoea*–*Erwinia* complex, although *L. rosea* and *P. strigatus* also harboured several other low‐abundance taxa. Fungal amplicon sequencing recovered low abundance taxa and unevenly represented communities, including *Cladosporium*, *Eremothecium* and unassigned Ascomycota, suggesting they were mostly environmentally acquired rather than conserved fungal associates. Despite host specificity, bacterial symbiont phylogeny was incongruent with host phylogeny, indicating host switches and decoupled host‐symbiont evolution. Egg‐surface sterilisation in *B. bibax* revealed vertical transmission of its dominant *Pantoea*‐like symbiont via egg smearing, and its role in host fitness, with aposymbiotic offspring showing reduced survival, delayed development, shorter longevity and lower fecundity. Overall, our findings extend established patterns of stink bug symbiosis to understudied Australian taxa, providing new insights into host–microbe associations, particularly for Tessaratomidae.

## Graphical abstract

For the first time, high‐throughput profiling of the crypt‐bearing M4 midgut region of four Australian stink bug species revealed host‐specific bacterial communities dominated by *Pantoea*–*Erwinia*‐related symbionts, contrasting with more variable, likely environmentally acquired fungal assemblages. In *Biprorulus bibax*, disrupting egg‐surface transmission of its dominant *Pantoea*‐like symbiont significantly reduced nymphal survival, delayed development, shortened adult longevity and lowered fecundity, highlighting the vital contribution of this microbial partnership to host fitness.

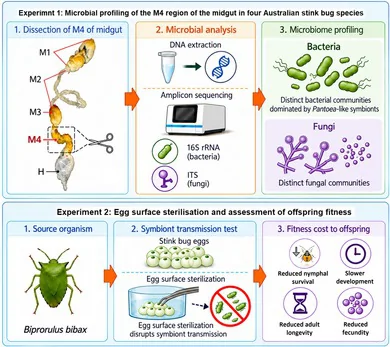

## 1 Introduction

Animal guts host diverse microbial communities, including bacteria and fungi that play key roles in host biology, ecology and evolution (Moran et al. 2019). These microbial symbionts can improve host nutrition by aiding the digestion and metabolism of complex or otherwise inaccessible dietary components (Rowland et al. 2018). They may also protect hosts by producing antimicrobial compounds or enhancing tolerance to stressors such as heat and chemical exposure (Rooks and Garrett 2016; Wang et al. 2024; Dearing and Weinstein 2022). In insects, especially those feeding on nutritionally restrictive diets (e.g., plant sap, blood and woody tissue), these interactions have driven evolution of intimate symbioses with specialised microbes that occupy distinct gut regions or specialised cells (e.g., bacteriocytes or mycetocytes), and these symbioses help overcome nutritional constraints and shape ecological interactions (Douglas 2015, 1989).

Among insects, gut microbial associations are well documented in Hemiptera, particularly Pentatomoidea (stink bugs and relatives; ~8000 species) (Duron and Noël 2016; Hosokawa, Ishii, et al. 2016; Çağatay et al. 2026). In many pentatomoids, the posterior midgut (M4) contains crypts densely packed with extracellular symbionts, usually dominated by a single, often host‐specific taxon of Enterobacteriaceae lineages (Bansal et al. 2014; Kikuchi 2009) that remain poorly characterised across numerous taxa. These bacteria can supplement limiting nutrients and facilitate host development, ecological performance and diversification (Duron and Noël 2016; Hosokawa, Ishii, et al. 2016; Moriyama and Fukatsu 2022; Kashkouli et al. 2021). In many stink bug species, gut symbionts are maternally transmitted, most often via egg smearing during oviposition, whereby newly hatched nymphs acquire symbionts by probing the eggshells (Hosokawa et al. 2013; Hosokawa et al. 2008). In some species, females deposit symbiont‐filled capsules beside the eggs that nymphs consume (Fukatsu and Hosokawa 2002). In contrast, in several pentatomids and other true bug lineages such as Coreoidea and Lygaeoidea, offspring acquire their gut symbionts horizontally from the environment in each generation (Nakawaki et al. 2024; Shan et al. 2021; Sullivan et al. 2025; Ravenscraft et al. 2026; Sullivan et al. 2026). Furthermore, while these gut bacterial associations are now recognised as being central to stink bug biology, their diversity and evolutionary patterns remain unresolved in many stink bug taxa.

Fungal microbiota, including yeast‐like symbionts, can also have major effects on insect physiology, development and nutrition (Kobiałka et al. 2018; Deng et al. 2026; Blackwell 2017; Hou et al. 2013), yet in hemipterans they have received far less attention than bacterial symbionts. In stink bugs, fungal diversity remains poorly characterised, with only a few available reports, which suggest that the fungal communities of some species are frequently dominated by ascomycete fungi, particularly *Cladosporium* (Li et al. 2024; Yang et al. 2023). Some species also vector plant‐pathogenic yeasts such as *Eremothecium coryli* which can cause stigmatomycosis in fruit (Foster and Daugherty 1969).

To date, relatively few studies have used tissue‐targeted high‐throughput amplicon sequencing focussing on the crypt‐bearing M4 midgut region (Bansal et al. 2014; Sun et al. 2026), and thus the overall diversity, including low‐abundance taxa, detected in dissected M4 tissue remains unresolved. This knowledge gap is particularly pronounced across Australian Pentatomoidea and, more globally, within the family Tessaratomidae, for which gut‐associated microbial communities remain generally poorly characterised (Kumari et al. 2024; Samal et al. 2025). Australia hosts > 400 pentatomoid species, and many of these are endemic; Pentatomidae and Tessaratomidae comprise > 65% of Australian Pentatomoidea and include several key sap‐sucking pests (Australian Biological Resources Study 2026). Australia is also at risk from incursions of exotic pentatomoids such as the brown marmorated stink bug ( *Halyomorpha halys* ), a highly invasive polyphagous species reported from a wide range of host plants; a recent incursion of *H. halys* in New South Wales during summer 2017–18 was declared eradicated in August 2018 (Horwood et al. 2019). To our knowledge, no previous study has comprehensively characterised the bacterial and fungal gut communities of native Australian stink bugs, and no study has experimentally tested the significance of their dominant gut symbionts for host fitness. This represents a crucial gap in the global understanding of pentatomoid diversity because studies in stink bugs have repeatedly shown that loss of key gut symbionts can impair development, survival, reproduction, and morphology (Prado and Almeida 2009; Karamipour et al. 2016; Hosokawa et al. 2007).

Here, we address this gap through a comparative analysis of gut microbial diversity and structure across four Australian stink bug species of two Pentatomoidea families collected from citrus trees: the spined citrus bug (SCB) *Biprorulus bibax* (Pentatomidae; citrus specialist), the gumtree shield bug (GSB) *Poecilometis strigatus* (Pentatomidae; primarily *Eucalyptus*‐associated but also feeds on citrus), the lychee stink bug (LSB) *Lyramorpha rosea* (Tessaratomidae; polyphagous on fruit trees) and the bronze orange bug (BOB) *Musgraveia sulciventris* (Tessaratomidae; specialised and major citrus pest) (Gross 1972; Cant et al. 1996; James 1989; Monteith 2011). These species were selected because they are four common Australian stink bug species representing two major pentatomoid families, include important pest species, and span a range of host specificity from citrus specialists to more generalists (Gross 1972; Cant et al. 1996; James 1989; Monteith 2011), while co‐occurring in the same environment that enables a more direct comparison of their gut microbial communities. Importantly, inclusion of two tessaratomid species allows us to extend comparative gut microbiome analyses to a family that remains poorly represented in the current stink bug symbiosis literature.

Using high‐throughput amplicon sequencing of the bacterial 16S rRNA gene and fungal ITS region, we characterised both bacterial and fungal communities associated with the midgut crypts (M4) of these species (Figure 1A–D). This represents, to our knowledge, the first integrated comparative characterisation of bacterial and fungal microbiota from the specialised M4 region of Australian stink bugs and one of the few such comparative studies in Pentatomoidea. We further strengthened this comparative survey with an experimental test of symbiont function in one of these four species, *B. bibax*. For this we established a laboratory population and used egg‐surface sterilisation to demonstrate the vertical transmission of this bug species' dominant gut symbiont and its contribution to host development, survival and fitness. We hypothesised that gut microbial diversity and community structure differ among stink bug species and between stink bug families, reflecting host evolutionary history and ecological strategy, and that dominant vertically transmitted symbionts are functionally important for host development and reproduction. Together, our study provides the first baseline for understanding bacterial and fungal gut symbioses in Australasian Pentatomoidea, particularly the globally poorly characterised Tessaratomidae, and advances understanding of the diversity, evolution and functional significance of host–microbe associations in stink bugs.

**FIGURE 1:**
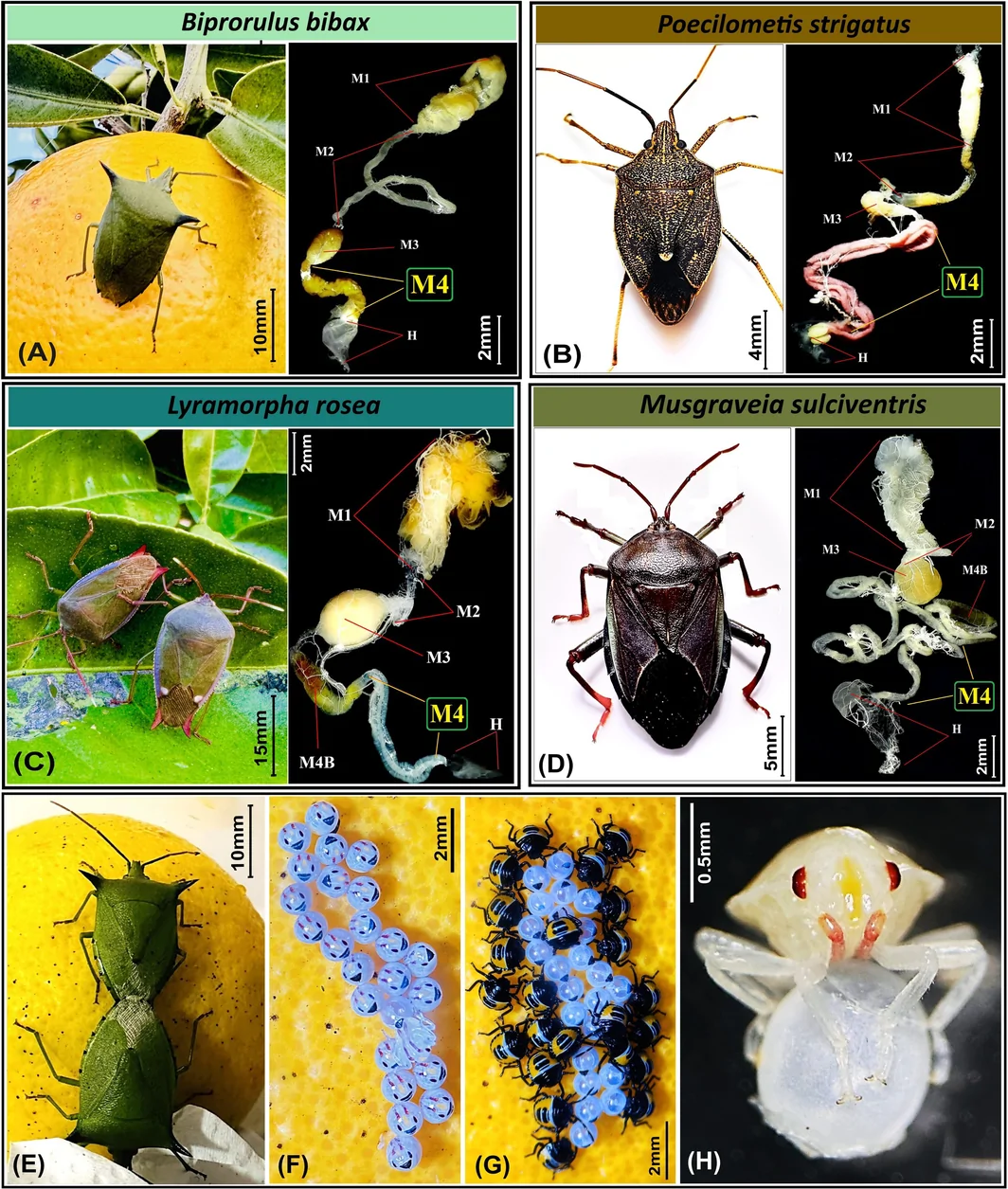
Study species, dissected gut region and spined citrus bug symbiont‐disruption assays. (A–D) Adult habitus (left) and dissected alimentary tract (right) of the four Australian stink bug species examined: (A) *Biprorulus bibax* (spined citrus bug, SCB, Pentatomidae), (B) *Poecilometis strigatus* (gumtree shield bug, GSB, Pentatomidae), (C) *Lyramorpha rosea* (lychee stink bug, LSB, Tessaratomidae) and (D) *Musgraveia sulciventris* (bronze orange bug, BOB, Tessaratomidae). Midgut regions are indicated as M1–M3 (anterior midgut), M4B (M4 bulb, where present) and M4 (posterior, crypt‐bearing midgut), followed by the hindgut (H). The M4 region (highlighted with an enlarged label in the box) was excised from the remainder of the gut for bacterial and fungal microbiota profiling. (E–H) Biology of *B. bibax* relevant to vertical symbiont transmission: (E) mating pair, (F) egg mass, (G) aggregation of newly hatched first‐instar nymphs around the egg mass and (H) close‐up of a newly hatched nymph showing typical probing behaviour on the eggshell surface.

## 2 Material and Methods

### 2.1 Experiment 1: Midgut Microbiota Profiling in Four Australian Stink Bug Species

#### 2.1.1 Gut Dissection and Sample Preparations

We characterised gut bacterial and fungal communities using high‐throughput amplicon sequencing of total genomic DNA extracted from the posterior midgut (M4 crypt region) of 32 individuals representing four species across two stink bug families: *B. bibax* (*n* = 10) and *P. strigatus* (*n* = 5) of the Pentatomidae, and *L. rosea* (*n* = 9) and *M. sulciventris* (*n* = 8 for 16S rRNA gene; *n* = 6 for fungal ITS) of the Tessaratomidae. Adult males and females were collected from citrus trees in an orchard of the Hawkesbury Campus, Western Sydney University in 2021 (33°36′36″ S, 150°44′47″ E; Table S1). Sampling was distributed across multiple spatially separated trees, with one individual collected per tree and trees spaced approximately 10 m apart within an area of about 1000 m^2^, to minimise close relatedness and direct contact among individuals. Specimens were freeze‐killed at −20°C for 24 h, surface‐sterilised (2% sodium hypochlorite for 2 min, Milli‐Q rinse, brief ethanol dip, air‐dried) and dissected in Ringer's solution (9.0 g NaCl, 0.42 g KCl, 0.37 g CaCl_2_ × 2H_2_O and 0.2 g NaHCO_3_ dissolved in 1 L of distilled water) using sterilised tools. The posterior midgut (M4 crypt region) was excised and transferred individually to sterile 1.5 mL tubes for DNA extraction.

#### 2.1.2 DNA Extraction From Gut Samples and Library Preparation for 16S rRNA Gene and ITS Amplicon Sequencing

The midgut (crypt‐bearing M4 region) samples were homogenised using a TissueLyser (Qiagen, Hilden, Germany) with one 2‐mm stainless steel ball per tube, operated at a frequency of 25 Hz for 4 min. Subsequently, DNA extractions were carried out using the Qiagen DNeasy Blood and Tissue kit as per the manufacturer's instructions, followed by an elution with 100 μL of nuclease‐free water. Extracted genomic DNA was quantified using fluorometry (Qubit, Thermofisher Scientific) and spectrophotometry (Nanodrop 2000, Thermofisher Scientific, Waltham, USA), and its integrity was verified by agarose electrophoresis. As an additional quality check, and to obtain host barcodes, we PCR‐amplified the mitochondrial cytochrome c oxidase I (COI) gene from each DNA extract using the universal primers LCO1490 and HCO2198 (Vrijenhoek 1994). The resulting PCR amplicons were Sanger sequenced, and the COI sequences generated for all four stink bug species were used for host COI barcoding and downstream host phylogenetic analyses. DNA extracts were stored at −20°C until library preparation and sequencing. DNA extracts were submitted to the Western Sydney University Next Generation Sequencing facility for high‐throughput amplicon sequencing using Illumina MiSeq (paired‐end 2 × 301 bp). For bacteria, the V3‐V4 region of the 16S rRNA gene was amplified using primers 341F (5′‐CCTACGGGNGGCWGCAG‐3′) and 805R (5′‐GACTACHVGGGTATCTAATCC‐3′) (Klindworth et al. 2013). For fungi, the ITS2 region was amplified using primers fITS7 (5′‐GTGARTCATCGAATCTTTG‐3′) and ITS4 (5′‐TCCTCCGCTTATTGATATGC‐3′) (Ihrmark et al. 2012). A bacterial mock community (ZymoBIOMICS Microbial Community DNA Standard) was included as a positive control.

#### 2.1.3 Bioinformatics

Raw sequencing reads were demultiplexed, and adapter sequences were removed with quality filtering based on Phred33 scores. The 16S rRNA gene and ITS amplicon datasets were analysed using USEARCH (Edgar 2010) combined with the Quantitative Insights Into Microbial Ecology 2 (QIIME2) framework (Bolyen et al. 2019). The quality filtering (trimming the low‐quality bases, *Q* value > 20) of raw reads was performed in USEARCH, and chimeric sequences were removed before denoising (error‐correction) of amplicon reads to generate the zOTU (zero‐radius operational taxonomic unit) using UNOISE (Edgar and Flyvbjerg 2015). The resultant zOTUs were assigned with taxonomic information in QIIME2 (version 2023.7) using a Naïve Bayes classifier trained on the SILVA (version 138) 16S rRNA gene database for bacterial 16S rRNA gene sequences and the UNITE v8.0 database for fungal ITS sequences (Quast et al. 2012; Abarenkov et al. 2024). Any bug‐associated mitochondrial, plant‐associated and chloroplast sequences were removed prior to downstream analyses. To assess whether the sequencing depth was sufficient, rarefaction curves were generated by plotting the number of observed microbial zOTUs against a range of sampling depths, averaged over 20 iterations. The curves reached a plateau for all samples, indicating that the sequencing depth was adequate and that the samples captured the majority of microbial diversity present (Figure S1).

Alpha diversity was estimated among the four stink bug species using the Shannon diversity index, and Simpson and Chao estimators. Values were compared using one‐way ANOVA (LSD test; *p* ≤ 0.05 was considered statistically significant), or the nonparametric Kruskal–Wallis test when the data were not normally distributed. The analyses were performed using SPSS software (v.27; IBM Corp, Chicago, USA).

Beta diversity was assessed using weighted UniFrac distance (phylogenetic relationships and relative abundance), unweighted UniFrac distance (phylogenetic relationships by presence/absence) and Bray–Curtis distance (relative abundance) to determine the microbial community difference in the four stink bug species. The effect of stink bug species and host range on gut microbial diversity and community composition were investigated using permutational multivariate analysis of variance (PERMANOVA, permutation = 9999), and visualised with principal component analysis (PCA) using the Vegan package (v.2.5‐6) (Oksanen et al. 2019) in R (v.4.0.3; R Core Team 2021, https://www.R‐project.org/). We further used ggplot2 package (version 3.3.3) in R to produce the stacked bar plots of gut bacteria and fungi at class and OTU level. For the analysis of the core microbiota (core OTUs), we used an online analysis tool (MicrobiomeAnalyst, https://www.microbiomeanalyst.ca/) following recommended parameters (Chong et al. 2020). Here, we computed the core microbiota of the four targeted stink bug species first using a 20% threshold, and subsequently we extended our analyses using a 40% and 60% threshold aiming to enhance the possibility of the detected gut microbiota to be biologically relevant to their hosts. We identified core bacterial populations (OTUs) as those present in at least half of the individuals within each host species or with a relative abundance greater than 1%.

#### 2.1.4 Bacterial and Fungal Co‐Occurrence

To assess potential associations among bacterial and fungal OTUs within each host species, host‐specific co‐occurrence networks were constructed in R v4.0.3 using pairwise Spearman's rank correlations based on OTU relative abundance data. Analyses were performed separately for each stink bug species using taxonomic abundance profiles derived from the amplicon sequencing data. Only statistically significant correlations (Spearman's correlation coefficients > 0.60, *p* < 0.05) were retained for network construction, with positive and negative correlations interpreted as putative co‐occurrence and co‐exclusion patterns, respectively (Faust and Raes 2012).

#### 2.1.5 Phylogenetic Analyses of the Dominant Bacteria

For phylogenetic analyses of the dominant bacterium in each host species, we further PCR‐amplified the near‐full‐length bacterial 16S rRNA gene (~1365 bp; V1–V9) using primers 16SA1 (5′‐AGAGTTTGATCMTGGCTCAG‐3′) and 16SB1 (5′‐TACGGYTACCTTGTTACGACTT‐3′) following the conditions described previously (Fukatsu and Nikoh 1998). This was performed on the genomic DNA extracts of dissected midguts (crypt‐bearing M4 region) from three individuals per stink bug species, selected from the genomic DNA extract samples used for MiSeq amplicon sequencing, as well as from two newly dissected individuals included in an additional round of Sanger sequencing to further confirm the results. Of the former, we selected genomic DNA extracts of midguts in which a single bacterial OTU dominated the community in the MiSeq dataset (70%–98% relative abundance in the OTU table) to minimise the impact of any other taxa on sequence analysis. Each PCR reaction was performed on the genomic DNA extracts of individuals (not pooled individuals). These PCR amplicons were then directly Sanger‐sequenced; forward and reverse chromatograms were manually inspected and showed clear peaks, with no evidence of double or overlapping peaks, ambiguous base calls or mixed‐template signals. Identical sequencing results were recovered across replicate individuals and in the additional sequences obtained from newly dissected material. Because the MiSeq amplicon data had already identified a highly dominant bacterial OTU in each selected sample, and direct Sanger sequencing consistently recovered a single clear sequence that included the same short amplicon sequence as obtained by MiSeq, molecular cloning was not considered necessary for recovery of the dominant symbiont sequence. When V3–V4 reads were aligned to the matching region of the V1–V9 16S rRNA gene sequences, the alignment exhibited 100% nucleotide identity across samples within bug species, matching the dominant MiSeq‐derived OTU in each bug species (Figure S2). The V1–V9 16S rRNA gene sequences (Figure S3) were aligned using ClustalX (Larkin et al. 2007). Phylogenetic inference was conducted on a trimmed 1365 bp alignment together with reference sequences of gut‐associated stink bug symbionts retrieved from NCBI based on BLAST‐based pairwise sequence similarities with 100% sequence coverage (1365 bp) across the aligned 16S rRNA gene region, which robustly resolved the placement of the identified core symbionts. Molecular phylogenetic analyses based on Maximum Likelihood (ML) and Neighbour Joining (NJ) algorithms were performed in MEGA v11.0.13. Maximum Likelihood used the GTR + G + I substitution model, selected as the best fit for the 16S rRNA gene dataset, whereas Neighbour‐Joining was performed using the Kimura 2‐parameter (K2P) distance model. Node support was assessed with 1000 bootstrap replicates for both methods. Phylogenetic trees were rooted with *Pseudomonas aeruginosa* (Pseudomonadales: Gammaproteobacteria) as a non‐Enterobacteriaceae outgroup.

To assess whether the phylogeny of the bacterial symbionts mirrored that of their stink bug hosts, we compared host phylogenies inferred from mitochondrial COI sequences (615 bp) (Figure S4) with corresponding symbiont phylogenies based on 16S rRNA gene sequences (V1‐V9) using a tanglegram approach. Five individual samples of each stink bug species were sequenced for COI. Because all sequences were identical within stink bug species, indicating no intraspecific variation, a single representative sequence per species was used for phylogenetic analysis. Maximum Likelihood (ML) trees for both host and symbiont datasets were reconstructed and visualised in Dendroscope software (version 3.8.10) (Huson and Scornavacca 2012). A tanglegram was generated to illustrate and evaluate topological congruence between host and symbiont trees. The degree of phylogenetic congruence was quantified using the I(cong) index, which tests whether the observed similarity between trees exceeds that expected by random association.

### 2.2 Experiment 2: Symbiont Elimination and Analysis of Fitness Parameters of *B. bibax*

A laboratory culture of *B. bibax* was established from 20 males and 20 females collected from the same citrus orchard during the austral winter (June 2024). Insects were maintained in mesh‐lidded containers (Katlav et al. 2021) at 25°C ± 1°C, 60% ± 5% RH and a 16:8 h light: dark photoperiod, and were provided with fresh oranges. Because adults collected in winter were reproductively dormant, females did not oviposit immediately; dormancy ended after 20–23 days. After oviposition commenced, 10 newly laid egg batches were collected. Egg masses were surface‐sterilised in 70% ethanol for 10 min, immersed in 4% sodium hypochlorite for 15 s, and rinsed twice with sterile water, whereas control egg masses were rinsed twice with sterile water only (Karamipour et al. 2016). DNA‐based PCR assays of a subset of eggs (five eggs per treatment) confirmed complete elimination of the dominant bacterial gut symbiont in treated eggs, but its presence in control eggs (not surface‐sterilised) based on 16S rRNA gene PCR using the V1–V9 primers. These results indicate that the ethanol–hypochlorite treatment effectively removed surface‐associated bacteria, including the dominant egg‐surface‐associated symbiont, prior to hatching.

To evaluate the effects of symbiont removal on host fitness, the development of individuals from 30 eggs per treatment was assessed individually. Egg hatching success was recorded for each egg as a binary response (hatched/unhatched). Hatchlings from each treatment were reared under the same laboratory conditions, and individual nymphs were monitored daily. This involved the assessment of survival to the adult stage, development time to adult, adult longevity (days from adult eclosion to death), and fecundity. To quantify fecundity, newly emerged adults were separated by sex, and individual females were paired with individual males (Figure 1E). Each pair was maintained in an individual container supplied with a fresh orange that had been surface‐sterilised in 1% bleach for 5 min and thoroughly rinsed with sterile water before being provided to the insects, thereby minimising opportunities for environmental acquisition of bacteria. Containers were inspected daily and all egg masses were collected and counted, with total fecundity calculated as the cumulative number of eggs produced per female over her lifespan. Egg hatching success and post‐hatching survival to the adult stage were analysed using binomial generalised linear models (GLMs). Continuous fitness traits, including egg‐to‐adult development time, adult longevity, and lifetime fecundity were compared between treatments using non‐parametric tests in SPSS, including Kruskal–Wallis and Mood's median tests.

## 3 Results

### 3.1 Amplicon Sequencing Overview

Using high‐throughput amplicon sequencing, we characterised the gut bacterial and fungal communities of four Australian native stink bug species. After quality control and filtering of the bacterial 16S rRNA gene amplicons, we obtained a total of 1,349,660 sequence reads (mean 42,177 reads per sample; range 2211–75,951), representing 476 OTUs clustered at 97% sequence similarity. For the fungal ITS dataset, quality filtering recovered 824 fungal OTUs. Because fungal sequencing depth varied substantially among samples, the ITS dataset was rarefied to 110 reads per sample prior to downstream diversity and community analyses to retain low‐depth samples (Figure S1).

### 3.2 Gut Bacterial Community Composition Across Stink Bug Species

At both the class and OTU levels, gut bacterial communities showed low overall diversity across all four host species but clear host‐associated differences in composition (Figure 2).

**FIGURE 2:**
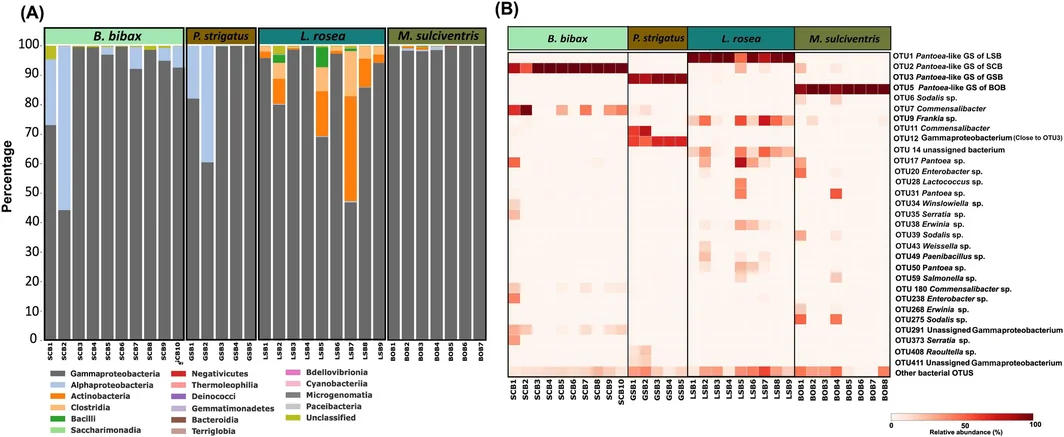
Bacterial community composition of the M4 crypt‐bearing midgut region of four Australian stink bugs species. (A) Stacked bar plots showing the relative abundance (%) of bacterial classes in individual bugs. (B) Heatmap of the dominant and prevalent bacterial OTUs (97% similarity OTUs) across the same individuals. Colour intensity indicates relative abundance (%) per sample.

#### 3.2.1 Biprorulus bibax

The bacterial community was dominated by Gammaproteobacteria (72%–100% relative abundance), except in SCB2 where Gammaproteobacteria were only ~44% and Alphaproteobacteria were ~56% (Figure 2A). At the OTU level, *B. bibax* was dominated by a *Pantoea*‐like symbiont (OTU2), a clear core member that accounted for 90%–99% of reads in most individuals, but only ~43%–50% in SCB1 and SCB2 (Figure 2B). Other taxa were generally low to moderate in relative representation, including *Commensalibacter* (OTU7; ~0.8%–2.5%) and an unclassified Gammaproteobacteria (OTU291; ~2.5%–55%). In SCB1, additional low‐abundance taxa were detected, including *Serratia* (OTU35 ~2.3%, OTU373 ~4.5%), *Enterobacter* (OTU238 ~6.5%) and another *Commensalibacter* (OTU180 ~1.2%) (Figure 2B).

#### 3.2.2 Poecilometis strigatus

Gut communities were dominated by Gammaproteobacteria (~60%–100%), with Alphaproteobacteria (20%–40%) detected mainly in GSB1 and GSB2 (Figure 2A). The dominant OTU was a *Pantoea*‐like symbiont (OTU3; 46.3%–75.2%). A second unassigned gammaproteobacterial OTU (OTU12), differing from OTU3 by a single nucleotide and closely related to it, was consistently present at moderate abundance (10.7%–24.1%), while *Commensalibacter* (OTU11) was prominent only in GSB1 and GSB2 (~18%–38%). Other taxa occurred at low abundance, generally between 0.4% and 1.4% (Figure 2B).

#### 3.2.3 Lyramorpha rosea

This species exhibited the highest bacterial diversity among the four stink bug species, although Gammaproteobacteria still dominated (~70.2%–99.8%), except in LSB6 where they were only ~45% (Figure 2A). Other consistently detected classes included Actinomycetes (2.1%–37.2%), Clostridia (2.3%–16.6%), Bacilli (0.2%–8.1%) and a range of unclassified taxa (0.1%–2.9%) (Figure 2A). At the OTU level, a *Pantoea*‐like symbiont (OTU1) dominated (46.3%–75.2%) the community. Additional abundant taxa included *Frankia* (OTU9; ~0.9%–34.2%) and an unassigned bacterium (OTU14; ~1.5%–12.5%). Several genera (*Lactococcus*, *Weissella*, *Kosakonia*, *Commensalibacter* and *Erwinia*) occurred sporadically and at trace levels (Figure 2B).

#### 3.2.4 Musgraveia sulciventris

The bacterial community was low‐diversity and strongly dominated by Gammaproteobacteria, primarily a *Pantoea*‐like symbiont (OTU5) accounting for 67.7%–99.9% of reads (Figure 2A). Other gammaproteobacterial taxa occurred sporadically, including *Kosakonia* (0.2%–8.5%), *Erwinia* (0.03%–9.4%), *Sodalis* (1.55%–9.4%) and *Frankia* (0.1%–0.6%). Other bacteria were detected only at trace levels (< 0.01%) (Figure 2B).

### 3.3 Gut Bacterial Community Diversity and Structure Across Host Species

Alpha diversity differed significantly among host species based on observed species, Chao1 richness and Shannon diversity index (Figure 3A–C). The bacterial community of *L. rosea* exhibited significantly higher observed species and Chao1 estimates than in *B. bibax* and *P. strigatus* (observed species: χ^2^ = 8.72, df = 3, *p* = 0.027; Chao1: χ^2^ = 9.15, df = 3, *p* = 0.033). Comparisons of Chao1 between *L. rosea* and *M. sulciventris* were marginally non‐significant but suggested a trend toward higher richness in *L. rosea* (observed species: *p* = 0.067; Chao1: *p* = 0.091). Shannon diversity also differed significantly among species (χ^2^ = 10.64, df = 3, *p* = 0.014), with *L. rosea* and *P. strigatus* showing similarly higher diversity than *B. bibax* and *M. sulciventris* (Figure 3A–C).

**FIGURE 3:**
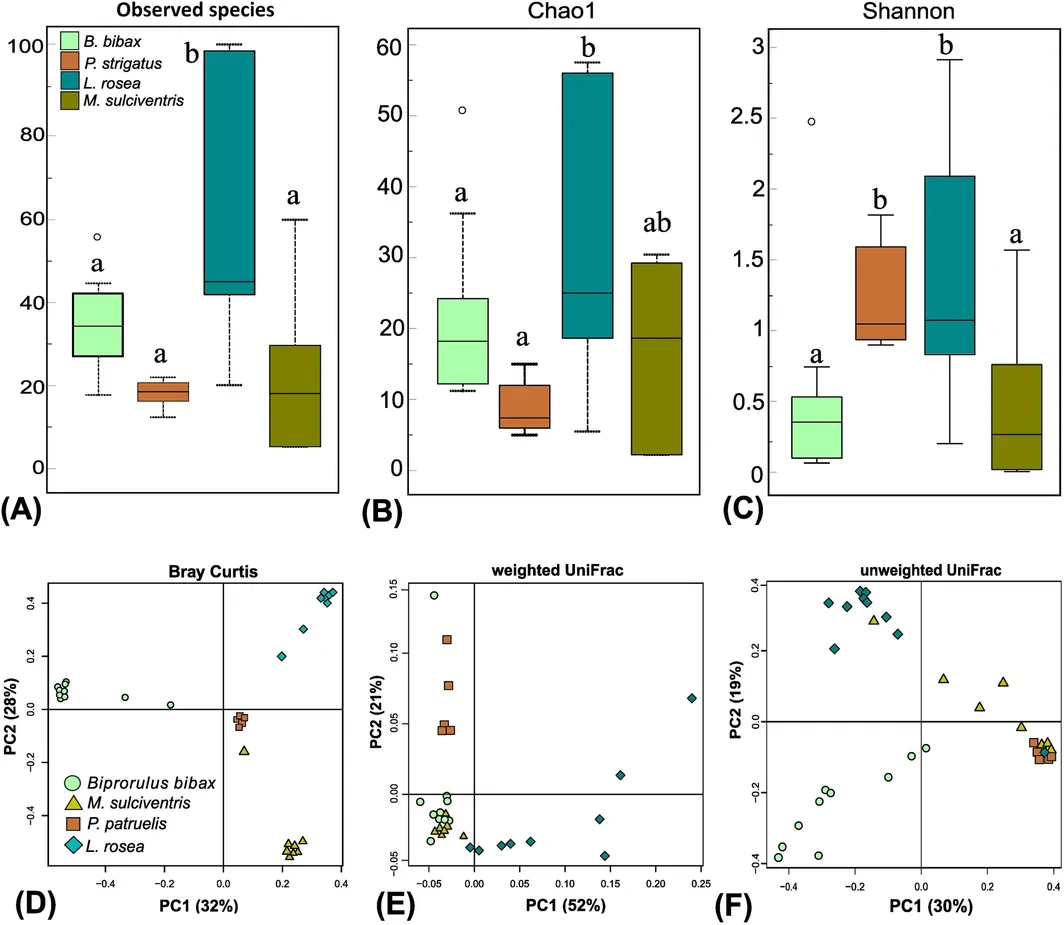
Alpha and beta diversity of bacterial communities in the crypt‐bearing M4 midgut region of four Australian stink bug species. (A–C) Alpha diversity metrics for individual bugs: (A) observed species richness, (B) Chao1 estimated richness and (C) Shannon diversity index. Boxplots show the median (line), interquartile range (box), and whiskers (1.5 × IQR), with outliers shown as points. Different letters denote significant pairwise differences among host species (Kruskal‐Wallis with post hoc comparisons; *p* ≤ 0.05). (D–F) Principal coordinate analysis ordinations of bacterial community composition based on (D) Bray–Curtis dissimilarity, (E) weighted UniFrac distance and (F) unweighted UniFrac distance. Points represent individual bugs coloured by host species; axis labels indicate the percentage of variation explained by each principal coordinate.

Beta diversity analyses (Bray–Curtis, weighted and unweighted UniFrac) showed clear host‐range clustering and significant differences in bacterial community composition among stink bug species (Figure 3D–F). PERMANOVA tests (999 permutations; Table 1) confirmed that bacterial communities differed significantly among all bug species, as well as between species within Pentatomidae and Tessaratomidae (Table 1). Significant differences were also detected between the two families and between host‐range groups (broad vs. narrow host plant specificity) (Table 1), indicating that both host phylogeny and ecological traits contribute to gut bacterial community structure.

**TABLE 1:** Summary of PERMANOVA results evaluating pairwise differences in beta diversity of gut bacterial (based on 16S rRNA gene sequence data) and fungal (based on ITS sequence data) communities in the crypt‐bearing M4 midgut region of four stink bug species using weighted UniFrac, Bray–Curtis and unweighted UniFrac distance metrics. Comparisons include: (i) among all four stink bug species; (ii) between pentatomid species (*Biprorulus bibax* and *Poecilometis strigatus*); (iii) between tessaratomid species ( *Lyramorpha rosea* and *Musgraveia sulciventris* ); (iv) between families (Pentatomidae vs. Tessaratomidae) and (v) between host range groups (narrow host range: *M. sulciventris* and *B. bibax* vs. broad host plant range: *L. rosea* and *P. strigatus* ).

Genes	PERMANOVA	*N*	Permutations	Weighted UniFrac		Bray–Curtis		Unweighted UniFrac	
				*F*	*P*	*F*	*P*	*F*	*P*
**16S rRNA**	All stinkbug species	32	999	11.11	**< 0.001**	32.14	**< 0.001**	6.24	**< 0.001**
	Between species in Pentatomidae	15	999	8.49	**0.004**	34.28	**0.002**	8.73	**< 0.001**
	Between species in Tessaratomidae	17	999	12.32	**0.003**	29.06	**< 0.001**	4.51	**0.004**
	Between families	32	999	7.24	**< 0.001**	11.51	**< 0.001**	4.39	**< 0.001**
	Between groups with different host plant range	32	999	9.25	**< 0.001**	10.88	**< 0.001**	2.96	**0.008**
**ITS**	All stinkbug species	38	999	4.01	**0.01**	2.53	**< 0.001**	3.26	**< 0.001**
	Between species in Pentatomidae	21	999	3.57	0.069	2.35	**< 0.001**	2.51	**0.013**
	Between species in Tessaratomidae	17	999	6.01	**0.013**	2.97	**< 0.001**	4.61	**< 0.001**
	Between families	38	999	3.43	**0.042**	2.13	**< 0.001**	2.94	**0.003**
	Between groups with different host plant range	38	999	0.88	0.337	2.82	**< 0.001**	3.11	**< 0.001**

*Note:* Comparisons that revealed significant differences are represented with a *p* value in bold.

### 3.4 Phylogenetic Placement of the Dominant Bacterial Symbionts and Cophylogenetic Analyses With Hosts

The AT content of the near full‐length 16S rRNA gene sequences (1365 bp) was 44.18% for the dominant midgut symbiont of *B. bibax*, 50.26% for *P. strigatus* , 48.79% for *L. rosea* and 47.13% for *M. sulciventris* . All midgut symbionts formed distinct lineages within Gammaproteobacteria but occupied different positions within the broader *Pantoea*‐*Erwinia* symbiont complex (Figure 4A). The *B. bibax* symbiont clustered closely with *Pantoea septica* (98.7% nucleotide identity across 1365 bp) and *Erwinia amylovora* (98.2%). The *P. strigatus* symbiont formed a more divergent lineage, branching separately and forming a sister lineage to symbionts of *Eurydema* species (97.3%) and *Niphe elongata* (97.1%) (Pentatomidae). The *M. sulciventris* symbiont clustered more basally relative to the *B. bibax* symbiont and grouped within an ancestral clade including the symbiont of *Nezara viridula* (Pentatomidae). The *L. rosea* symbiont grouped within a moderately supported clade of pentatomoid‐associated symbionts including *Graphosoma lineatum* (97.9%) and *Erthesina fullo* (97.6%) (Pentatomidae) (Figure 4A). The percentage identity values shown in parentheses represent BLAST‐based pairwise sequence similarities and are reported only for matches with 100% sequence coverage (1365 bp) across the aligned 16S rRNA gene region used for phylogenetic analysis.

**FIGURE 4:**
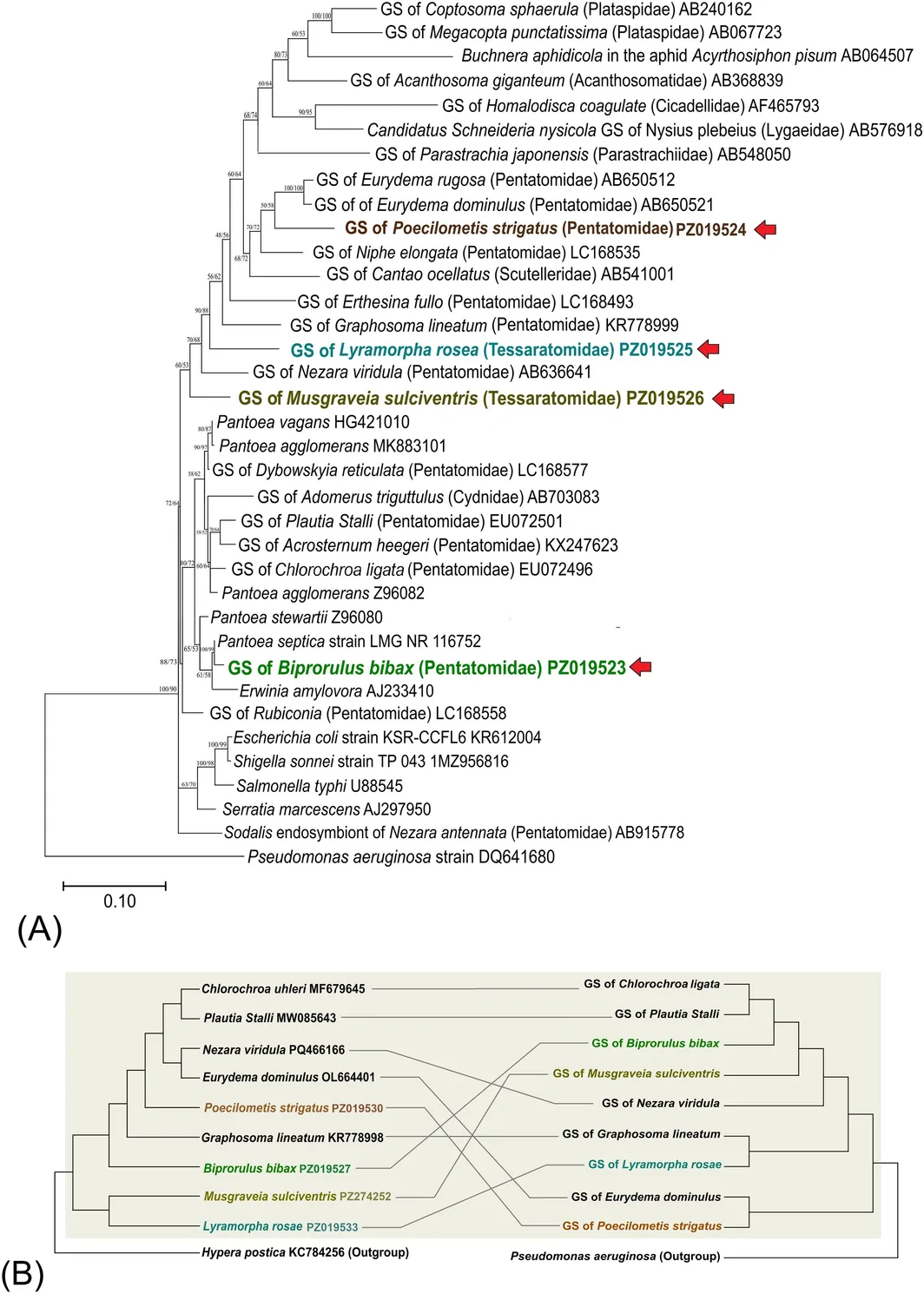
Phylogenetic placement of dominant gut symbionts and host‐symbiont cophylogeny. (A) Phylogeny based on near full‐length 16S rRNA gene sequences (1365 bp) showing the placement of the dominant gut symbionts (GS) from four stink bug species (five replicates were analysed per stink bug species; only one representative sequence is shown because all sequences were identical within each species) within the *Pantoea–Erwinia* complex. Numbers at nodes indicate Maximum Likelihood/Neighbour Joining bootstrap support (1000 replicates), and the scale bar shows substitutions per site. Additional bacterial phylogeny based on MiSeq amplicon sequencing data from the V3–V4 region is presented in Figure S6. (B) Tanglegram comparing host cytochrome c oxidase I (COI; 615 bp) phylogeny (left) and symbiont 16S rRNA gene (1365 bp) phylogeny (right). Dotted lines link hosts to their GS; crossings indicate topological incongruence, consistent with no strict host‐symbiont cospeciation. Five COI sequences were obtained per stink bug species; as all were identical within each species, one representative sequence per species was used for phylogenetic analysis. An additional host COI phylogeny across different stink bug taxa is provided in Figure S7.

Cophylogenetic analyses using host mitochondrial COI and symbiont 16S rRNA gene sequences showed no significant congruence between host and symbiont lineages (Figure 4B). Multiple mismatched branching patterns suggested largely independent evolutionary histories. The I_cong_ congruence index confirmed that the topologies were not more similar than expected by chance (*p* = 0.85), which may indicate host switching and/or symbiont replacement during diversification.

### 3.5 Gut Fungal Community Composition Across Stink Bug Species

Fungal community composition in the M4 midgut crypt region showed high within‐species variability, yet each stink bug species displayed a distinct overall fungal community profile (Figure 5).

**FIGURE 5:**
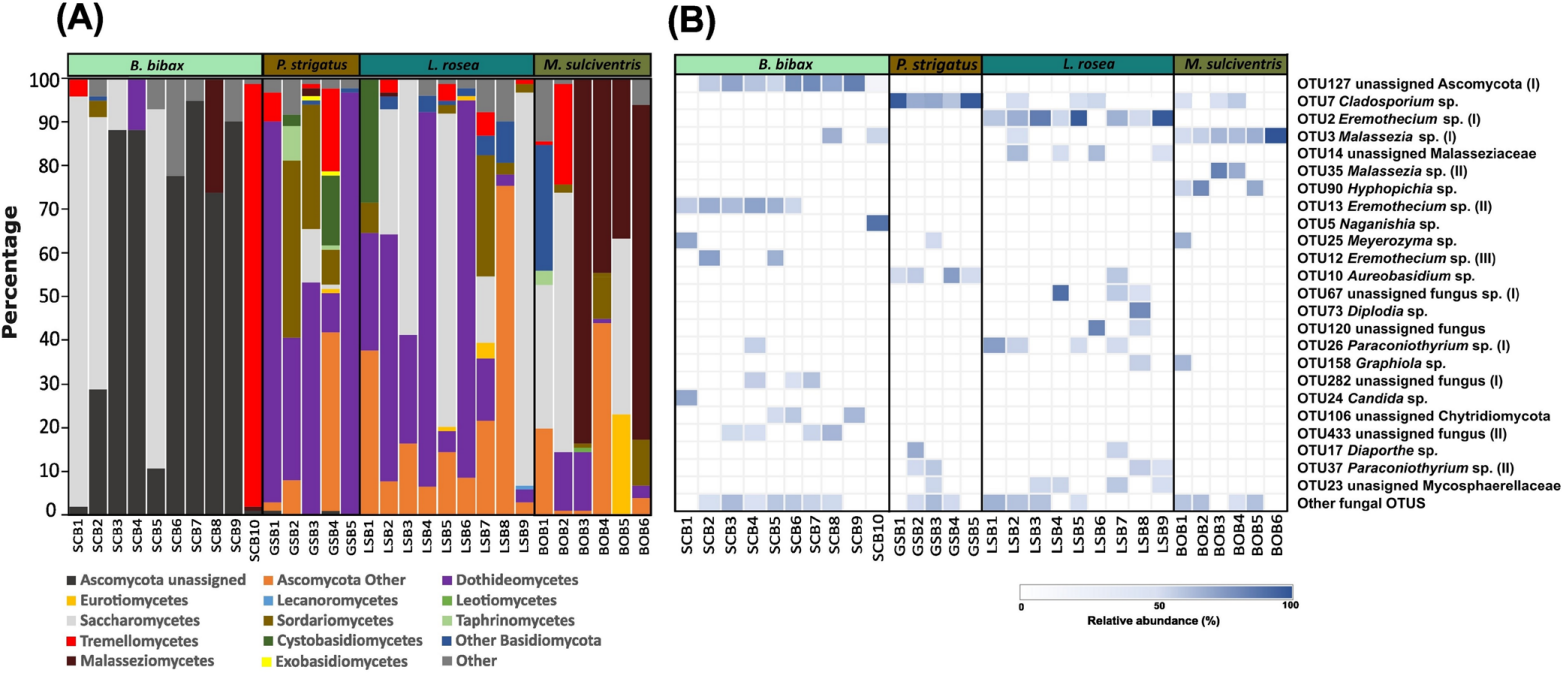
Fungal community composition of the M4 crypt‐bearing midgut region of four Australian stink bug species. (A) Stacked bar plots showing the relative abundance (%) of fungal classes in individual bugs. (B) Heatmap showing the dominant and prevalent fungal operational taxonomic units (clustered at 97% sequence similarity) across the same individuals. Colour intensity indicates the relative abundance (%) of each operational taxonomic unit per sample.

#### 3.5.1 Biprorulus bibax

The fungal community showed consistently low diversity but substantial individual variation, typically dominated by an unclassified Ascomycota (~70%–90%) (Figure 5A). In SC1, SC2 and SC5, its proportion dropped below 30%, with Saccharomycetes becoming more prominent, while SCF10 was dominated by Tremellomycetes (~95%), representing a clear outlier (Figure 5A). At the OTU level, core members included an unassigned Ascomycota (OTU127) and *Eremothecium* spp. (OTU12, OTU13), together contributing up to ~70% across individuals (Figure 5B). Other taxa (e.g., *Naganishia* sp. OTU5, dominant in SCB10 > 80%; *Malassezia* sp. OTU3; unassigned Chytridiomycota OTU106) were sporadic and did not form a consistent core (Figure 5B).

#### 3.5.2 Poecilometis strigatus

The fungal community was primarily dominated by Dothideomycetes (10%–95%), with Sordariomycetes contributing substantially in some individuals (up to 40%). Other fungal classes (e.g., Cystobasidiomycetes, Tremellomycetes, other Ascomycota) occurred sporadically and at lower abundance (Figure 5A). At the OTU level, *Cladosporium* sp. (OTU7) was consistently present (10%–95%) and appeared as a dominant and potential core member, while *Aureobasidium* sp. (OTU10) reached up to 40% in some individuals (Figure 5B). Additional taxa (e.g., *Diaporthe* sp. OTU17, *Paraconiothyrium* sp. (II) OTU37, *Meyerozyma* sp. OTU25) occurred at low abundance in subsets of samples (Figure 5B).

#### 3.5.3 Lyramorpha rosea

The fungal community varied across individuals but was typically dominated by Dothideomycetes and an unclassified Ascomycota, together comprising ~20% to > 90% of abundance. Saccharomycetes also occurred at moderate to high levels in approximately half of individuals (~20% to > 70%). Other classes (Sordariomycetes, Cystobasidiomycetes, Tremellomycetes and Eurotiomycetes) were sporadic and generally low abundance (Figure 5A). At the OTU level, *Eremothecium* sp. (I) (OTU2) dominated (up to ~90%), with *Paraconiothyrium* sp. (I) (OTU26; ~4%–48%) and additional taxa (unassigned Mycosphaerellaceae OTU23 ~9%; Malasseziaceae OTU14 ~2.6%) detected in some individuals (Figure 5B).

#### 3.5.4 Musgraveia sulciventris

The gut fungal community varied among individuals but was dominated by Malasseziomycetes, with highly variable relative abundance (~10%–80%) (Figure 5A). Sordariomycetes (0%–50%) and unclassified Ascomycota (0%–40%) also contributed substantially across samples (Figure 5A). Other classes (Exobasidiomycetes, Tremellomycetes and Dothideomycetes) occurred sporadically and generally at low to moderate abundance (Figure 5A). At the OTU level, *Malassezia* sp. 1 (OTU3) was detected in all individuals, while *Malassezia* sp. 2 (OTU35) occurred only in BOB3 and BOB4 (Figure 5B). Additional taxa, including *Hyphopichia* sp. (OTU90) and *Cladosporium* sp. (OTU7), were present at low abundance in some individuals (Figure 5B).

### 3.6 Gut Fungal Community Diversity and Structure Across Host Species

Alpha diversity analyses showed no significant differences in fungal community richness or diversity among the four stink bug species (Figure 6A–C): observed species (χ^2^ = 4.26, df = 3, *p* = 0.235), Chao1 richness (χ^2^ = 3.84, df = 3, *p* = 0.279) and Shannon diversity (χ^2^ = 5.19, df = 3, *p* = 0.158). Nevertheless, within‐species variation was substantial, with *M. sulciventris* showing the lowest variability (observed species = 10.4 ± 3.3; Chao1 = 13.1 ± 4.8; Shannon = 1.7 ± 0.4), indicating a consistently depauperate and uniform fungal community, whereas *P. strigatus* showed the highest variability in Chao1 and Shannon (Chao1 = 30.7 ± 12.2; Shannon = 3.3 ± 1.0).

**FIGURE 6:**
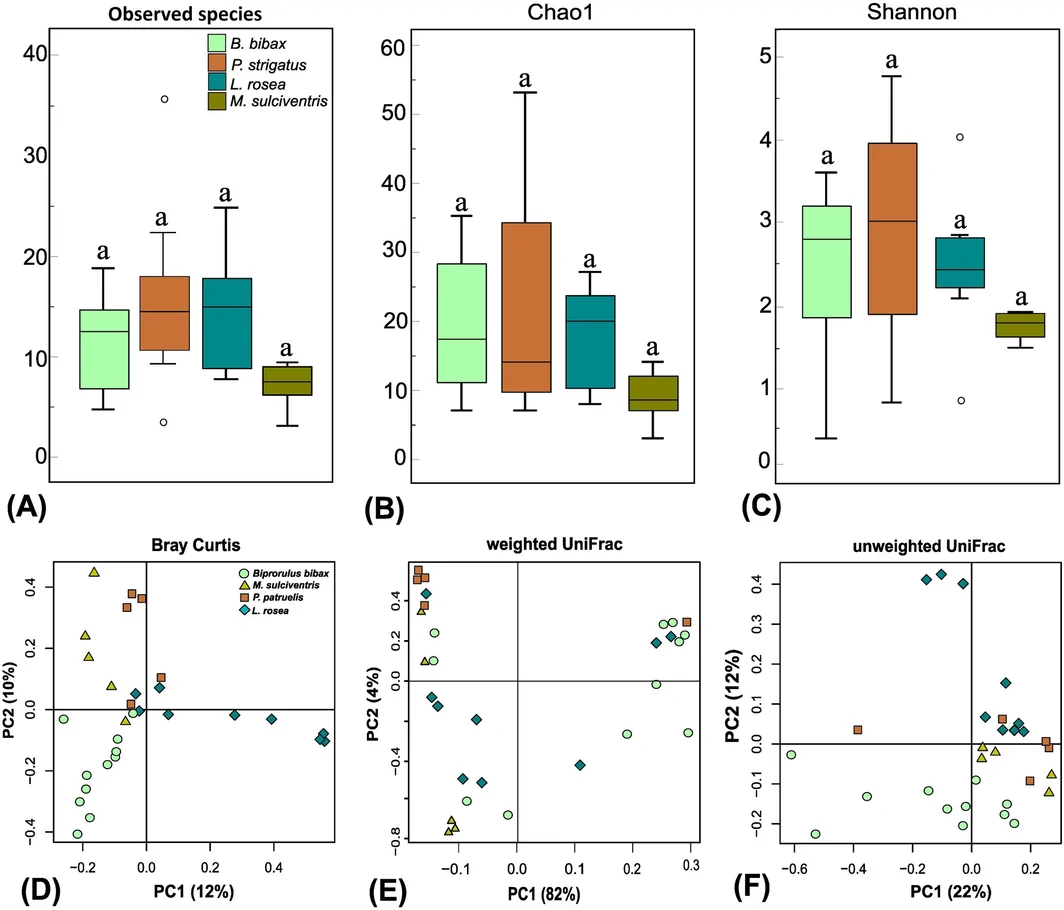
Alpha and beta diversity of fungal communities in the crypt‐bearing M4 midgut region of four Australian stink bug species. (A–C) Alpha diversity metrics for individual bug samples: (A) observed species richness, (B) Chao1 estimated richness and (C) Shannon diversity index. Boxplots show the median (line), interquartile range (box), and whiskers (1.5 × IQR), with outliers shown as points. Letters indicate results of post hoc comparisons; no significant differences were detected among host species (Kruskal–Wallis test; *p* > 0.05). (D–F) Principal coordinate analysis ordinations of fungal community composition based on (D) Bray–Curtis dissimilarity, (E) weighted UniFrac distance and (F) unweighted UniFrac distance. Points represent individual bugs coloured by host species; axis labels indicate the percentage of variation explained by each principal coordinate.

Beta diversity analyses (Bray–Curtis, weighted and unweighted UniFrac) revealed significant differences in fungal community composition among host species (Figure 6D–F; Table 1). PERMANOVA tests (999 permutations) supported overall host‐associated structuring. Fungal assemblages differed more strongly among the two Tessaratomidae species (*F* = 2.97–6.01, *p* ≤ 0.013) than among the two Pentatomidae species (*F* = 2.35–3.57, *p* = 0.013–0.069), while between‐family and host‐range comparisons were also significant for most metrics (Table 1). Together, these results indicate that gut fungal communities vary with host taxonomy and ecological traits, with stronger divergence within Tessaratomidae and among species with different plant host‐range.

### 3.7 Bacterial and Fungal Co‐Occurrence

Host‐specific co‐occurrence network analysis further revealed differences in the pattern and complexity of associations among selected bacterial and fungal OTUs across the four stink bug species. The co‐occurrence networks appeared to be simpler for the two citrus specialists *B. bibax* and *M. sulciventris* than the more generalist *P. strigatus* and *L. rosea*. However, no clear co‐occurrence pattern of the *Pantoea*‐like symbionts with particular fungal symbionts emerged across the four stink bug species (Supporting Information, Figure S5A–D).

### 3.8 Gut Symbiont Elimination Negatively Impacted *B. bibax* Fitness

Egg‐surface sterilisation successfully eliminated the *Pantoea*‐like gut symbiont from *B. bibax* eggs and markedly affected the biological performance of the offspring. Although egg hatching success remained high and did not differ significantly between treatments (binomial GLM: *z*_(1, 58)_ = −0.46, *p* = 0.64), with 93.3% hatching in the control group and 90.4% in the aposymbiotic group (Figure 7A), post‐hatching survival to the adult stage was significantly lower in aposymbiotic individuals (binomial GLM: *z*_(1, 53)_ = −2.18, *p* = 0.029), declining from 89.3% in the control group to 63.1% in the aposymbiotic group, representing a 29.45% reduction relative to the control (Figure 7B). However, the individuals that survived to the adult stage showed prolonged egg‐to‐adult development time compared with the control group (*χ* ^2^_(1,42)_ = 19.99, *p* < 0.001; Figure 7C), taking on average 51.3 ± 2.8 days versus 43.2 ± 1.7 days in controls. In addition, individuals in the aposymbiotic treatment were noticeably smaller in size although no other morphological abnormalities were observed. Adult longevity was also substantially reduced in aposymbiotic insects (*χ* ^2^_(1,42)_ = 16.72, *p* < 0.001; Figure 7D), averaging 29.4 ± 3.1 days compared with 48.1 ± 2.5 days in symbiotic controls. Likewise, total fecundity (number of eggs per female during life time) was significantly lower in aposymbiotic females (*χ* ^2^_(1,21)_ = 13.20, *p* < 0.001; Figure 7E), with a mean of 44.6 ± 6.2 eggs compared with 77.8 ± 7.1 eggs in controls.

**FIGURE 7:**
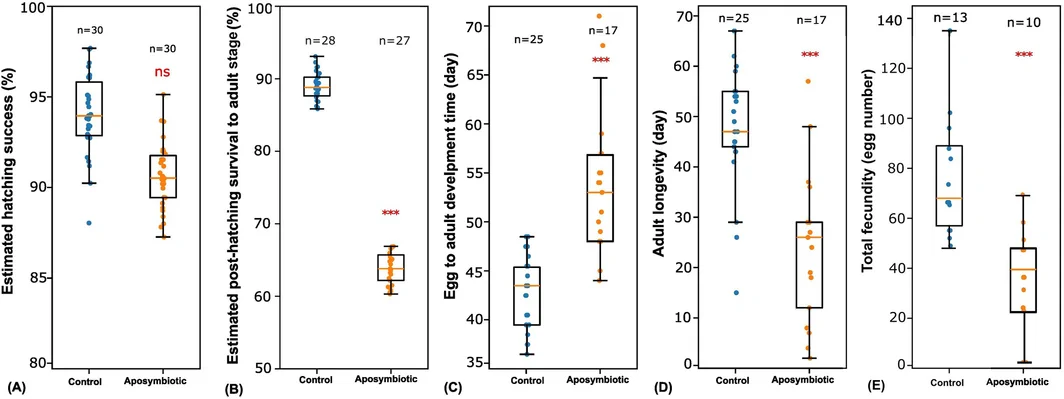
Fitness effects of elimination of the dominant midgut symbiont in *Biprorulus bibax*. Boxplots compare control (symbiotic) and aposymbiotic individuals produced by egg‐surface sterilisation. (A) Estimated hatching success (%), (B) estimated post‐hatching survival to adult stage (%), (C) development time from egg to adult (days), (D) adult longevity (days) and (E) total fecundity (egg number) in control and aposymbiotic individuals. Points represent individual replicates, boxplots show median and interquartile range, and whiskers indicate variability. Sample sizes are indicated above each group. Significant differences between treatments are indicated by asterisks (***p < 0.001), while ns denotes non‐significant differences.

## 4 Discussion

Our study provides the first comprehensive characterisation of gut microbial communities in four Australian native stink bug species: *B. bibax* and *P. strigatus* (Pentatomidae), and *L. rosea* and *M. sulciventris* (Tessaratomidae) (Figure 1A–D). By simultaneously characterising bacterial and fungal communities from the specialised M4 crypt‐bearing midgut region and including two tessaratomid species, our study fills a major gap in both the Australian Pentatomoidea fauna and the globally understudied Tessaratomidae. These findings establish a new baseline for comparative studies of stink bug microbiota and host–microbe evolution in Australasian taxa.

### 4.1 Low‐Diversity Gut Bacterial Communities Dominated by Host‐Specific *Pantoea*‐Like Symbionts in Four Australian Stink Bug Species

Across all species, bacterial diversity in the crypt‐bearing M4 midgut region was consistently low and dominated by Gammaproteobacteria, driven in each stink bug species by a single host‐specific OTU affiliated with the *Pantoea*‐*Erwinia* complex (Enterobacteriaceae), as is typical of pentatomoid midgut‐crypt symbioses (Kashkouli et al. 2021; Karamipour et al. 2016; Shan et al. 2021; Çağatay et al. 2026). Communities were most streamlined in the citrus specialists *B. bibax* and *M. sulciventris* , where the dominant *Pantoea*‐like OTUs (OTU2 and OTU5, respectively) comprised > 90% of reads. In contrast, *L. rosea* showed greater inter‐individual variation and measurable contributions from non‐proteobacterial classes (Actinomycetes and Bacilli), including *Frankia* and sporadic low‐abundance genera (*Lactococcus*, *Weissella*, *Kosakonia*, *Commensalibacter* and *Erwinia*). Furthermore, *P. strigatus* also consistently harboured, in addition to the dominant core *Pantoea*‐like bacterium OTU3, a second core gammaproteobacterial OTU12 at moderate abundance across all samples. OTU12 differed from OTU3 by only a single nucleotide in the V3–V4 region and clustered within the same branch in the phylogeny (Figure S6), suggesting a very closely related co‐occurring variant. However, the near full‐length 16S rRNA gene sequence (V1–V9) recovered by Sanger sequencing matched only OTU3, and further work is needed to determine how diverged OTU12 is from OTU3 in other genome regions.

Based on the alpha diversity metrics, *L. rosea* showed significantly higher richness (observed species and Chao1) than *B. bibax* and *M. sulciventris* , and Shannon diversity was higher in *L. rosea* and *P. strigatus* , indicating more even communities. Beta diversity ordinations separated the four species into distinct clusters, and PERMANOVA confirmed significant differences among species, between families and between host‐range groups (narrow vs broad). Together, this suggests that while a conserved *Pantoea*‐*Erwinia* complex symbiosis underpins community structure across these pentatomoid bugs, variation in richness and composition is further shaped by host ecology via environmental bacteria. This aligns with the broader host plant ranges of *P. strigatus* (Rutaceae, Myrtaceae, Proteaceae, Rosaceae and Oleaceae) (Gross 1972) and *L. rosea* (Sapindaceae and Rutaceae) (Monteith 2011), compared with the citrus specialists *B. bibax* and *M. sulciventris* (Hosokawa et al. 2007; Gross 1972). Broader host range of an insect likely increases exposure to diverse microenvironments, diets and plant compounds (including antimicrobials), and may increase opportunities for horizontal acquisition, collectively promoting higher secondary microbial diversity (Brunetti et al. 2022; Ge et al. 2025; Hosokawa, Matsuura, et al. 2016). However, this inference remains tentative because it is based on currently available host plant range information, and our sampling design did not allow the effects of host plant range, tree identity or microhabitat to be separated from bug taxonomic identity.

### 4.2 Phylogenetic Placement of Gut Bacterial Symbionts and Host‐Symbiont Coevolution

Bacterial symbionts of pentatomoids are generally placed within plant‐associated *Pantoea* and *Erwinia* lineages, but without clear evidence of strict host–symbiont co‐evolution (Karamipour et al. 2016). Our near full‐length 16S rRNA gene phylogeny showed that the dominant gut symbionts of all four Australian stink bug species fall within the broad gammaproteobacterial *Pantoea–Erwinia* complex, yet each host species harboured a distinct lineage: the symbiont of *B. bibax* clustered near *Pantoea septica* and *Erwinia amylovora* ; the symbiont of *P. strigatus* grouped with those of *Eurydema* spp. and *N. elongata* ; the symbiont of *L. rosea* with those of *G. lineatum* and *Erthesina fullo*; and the symbiont of *M. sulciventris* more basally near that of *N. viridula* . Together, these results support a conserved pentatomoid symbiosis type, with Enterobacteriaceae dominating the crypt‐bearing posterior midgut but diversified into host species‐specific lineages (Karamipour et al. 2016; Shan et al. 2021). Consistent with this, cophylogenetic analysis showed no significant congruence between host COI and symbiont 16S rRNA gene phylogenies (Figure 3B), arguing against long‐term strict co‐speciation and instead supporting evolutionary turnover and/or occasional replacement among closely related lineages adapted to the stink bug midgut crypt environment (Hosokawa, Matsuura, et al. 2016). Such turnover may be facilitated by extracellular transmission via egg‐associated inocula, allowing replacement while maintaining a similar functional niche (Larkin et al. 2007; Huson and Scornavacca 2012).

### 4.3 Variable, Low‐Abundance Fungal Communities in Stink Bug Midgut Crypts Suggest Environmentally Influenced Associations

Across the four Australian stink bug species, fungal communities in the crypt‐bearing M4 midgut region were more variable than bacterial communities and lacked a single dominant symbiont. This is biologically plausible, as the crypt‐bearing midgut region is primarily known as a bacterial symbiont‐bearing tissue; fungi in this region may therefore be low‐abundance, transient, environmentally acquired or more abundant in other gut regions. Yeast‐like and other plant‐associated fungi often form flexible, environmentally driven associations with insects, ranging from transient passengers to ecologically important partners (Stefanini 2018; Malassigné et al. 2021). Nevertheless, the recurrent detection of some fungal taxa may indicate ecologically meaningful associations. *Eremothecium* OTUs, particularly in *B. bibax* and *L. rosea* , are well known from piercing‐sucking insects such as Hemiptera and have been associated with stink bug‐mediated transmission and yeast‐spot plant diseases (Foster and Daugherty 1969; Kimura et al. 2008). Similarly, Dothideomycetes‐dominated profiles, including *Cladosporium* and *Aureobasidium* in *P. strigatus* and *L. rosea* , are consistent with the common occurrence of these fungi in the phyllosphere and as plant endophytes that insects may ingest during feeding (Kimura et al. 2008; Sword et al. 2017). Thus, while our fungal dataset should be interpreted cautiously because of low and uneven ITS read depth, it suggests that fungal communities associated with stink bug midgut crypts are mostly host‐variable, environmentally influenced and dominated by yeast‐like or plant‐associated taxa rather than conserved fungal symbionts.

Although fungal alpha diversity did not differ significantly among species, within‐species variation was high and beta diversity showed host‐associated structuring (including stronger divergence within Tessaratomidae and host plant‐range effects). This combination, similar overall richness but different composition, is consistent with a scenario where gut fungal communities are shaped by host taxonomy and ecology (diet breadth, feeding microhabitat, exposure to plant‐associated fungi), but do not form a uniformly conserved core across individuals in the way that midgut‐crypt bacteria do (Stefanini 2018). Although some bacteria‐fungi co‐occurrence patterns were detected within individual stink bug species, no consistent association was found between the dominant *Pantoea*‐like symbionts and any particular fungal taxon across all four species. This suggests that fungal associations may not be as central to host biology as the bacterial symbionts, or that any functional role of fungi may be carried out by a wider range of phylogenetically diverse taxa rather than by a single consistent fungal partner. Furthermore, the amplicon sequencing read return for fungi was substantially lower than for bacteria, which could further indicate that fungal taxa detected in the midgut of the studied bug species are more of a transient nature. Therefore, more research is required to determine whether any fungal taxa are consistently associated with the crypt‐bearing M4 midgut region within and across stink bug species. Future work should prioritise identifying such core fungi, quantifying their abundance with targeted methods, and resolving their spatial localisation within the gut using tissue‐specific and microscopy‐based approaches. These steps will be essential for distinguishing persistent associates from transient diet‐ or environment‐derived fungi and for establishing a stronger basis for functional investigation.

### 4.4 Fitness Role of Gut Symbiont in *B. bibax*

In *B. bibax* (Figure 1E), newly hatched nymphs remained aggregated around the eggshell for ~2 days (Figure 1G,H), repeatedly probing the egg surface, likely to acquire the maternally deposited *Pantoea*‐like symbiont. Egg‐surface sterilisation eliminated this symbiont and produced aposymbiotic offspring, confirming vertical transmission via egg smearing, a common mechanism in stink bugs with midgut‐crypt symbionts, where disruption typically reduces host performance (Gonella et al. 2020). Although these results strongly support vertical transmission, environmental acquisition cannot be completely excluded. The *B. bibax* symbiont is phylogenetically close to free‐living *Pantoea*/*Erwinia*‐related bacteria, suggesting that it may retain some capacity for environmental persistence or acquisition. Because the fruit supplied during our experiment was surface‐sterilised to minimise environmental bacterial exposure, opportunities for natural symbiont reacquisition may also have been reduced. Whether the symbiont remains culturable outside the host and whether environmental acquisition contributes to transmission under natural conditions should therefore be examined in future studies.

Consistent with this, aposymbiotic *B. bibax* were generally smaller in size (personal observation), showed ~30% lower survival, developed more slowly (~8‐day delay), had reduced adult longevity (~19 days shorter), and females laid ~40% fewer eggs than controls. Similar fitness costs have been reported after symbiont‐disruption in other stink bugs, including *G. lineatum* , *Eurygaster integriceps* and *H. halys* (Karamipour et al. 2016; Kafil et al. 2013; Taylor et al. 2014). These effects are plausibly driven by nutritional constraints in sap‐feeding bugs, where symbionts can supplement limiting nutrients; in *H. halys* , genomic evidence supports such roles for “Candidatus *Pantoea carbekii*” (Kenyon et al. 2015). Overall, our results place *B. bibax* among pentatomids with strong dependence on extracellular gut symbionts, consistent with variation in symbiont reliance across stink bug lineages (Gonella et al. 2020). Future work should test fitness effects in the other stink bug species, resolve symbiont function using genome‐ and/or metabolite‐based approaches, and determine transmission routes by tracking symbionts across life stages. In several Tessaratomidae species, females guard egg masses and first‐instar nymphs, and the newly hatched nymphs are extremely flattened and reportedly non‐feeding at the first instar (Monteith 2011). It will therefore be important to test whether maternal care behaviours facilitate vertical transmission of gut symbionts (e.g., by maintaining close contact with eggs/nymphs surfaces or maternal secretions) in *L. rosea* and *M. sulciventris* .

## 5 Conclusion

Overall, our findings provide new insight into gut symbiosis in the poorly characterised Australian Pentatomoidea fauna and broaden current understanding of microbial associations in the globally understudied Tessaratomidae. We found that the four studied native Australian stink bug species harbour low‐diversity gut bacterial communities in the crypt‐bearing M4 midgut region dominated by host‐specific *Pantoea*‐*Erwinia* complex symbionts. Phylogenetic and cophylogenetic analyses identified host‐associated symbiont lineages but no strict host–symbiont co‐speciation, indicating evolutionary turnover and partly decoupled host–symbiont evolutionary histories. In *B. bibax*, disrupting maternal transmission produced aposymbiotic offspring with reduced fitness, demonstrating the functional importance of the dominant bacterial symbiont. In contrast, low‐abundance bacteria and fungi were more variable among host species, with patterns likely influenced by ecological differences such as host‐plant range. These microbial taxa may represent true but rare associates, transient microbes, or contamination from adjacent gut tissues or gut contents. Broader sampling across regions, seasons, host plants and life stages, together with localisation‐ and quantification‐based analyses such as FISH and qPCR will therefore be needed to test the robustness of these patterns and move beyond amplicon‐based snapshots toward a more complete understanding of gut microbial dynamics in Australian true bugs.

## Author Contributions

**Alihan Katlav:** conceptualization, methodology, software, data curation, investigation, validation, formal analysis, funding acquisition, visualization, project administration, writing – original draft, writing – review and editing. **Juntao Wang:** software, validation, formal analysis, writing – review and editing. **Piotr Trębicki:** funding acquisition, conceptualization, methodology, writing – review and editing, supervision. **Amir H. Tourani:** methodology, resources, investigation. **Jennifer L. Morrow:** formal analysis, software, writing – review and editing, visualization. **Hongwei Liu:** software, visualization, writing – review and editing. **Markus Riegler:** conceptualization, methodology, software, validation, supervision, funding acquisition, project administration, resources, writing – original draft, writing – review and editing.

## Funding

This work was supported by the Australian Postgraduate Research Intern (APR.Intern) program (INT‐1050).

## Conflicts of Interest

The authors declare no conflicts of interest.

## Supporting information


**Figure S1:** Rarefaction curves of bacterial and fungal communities. Rarefaction plots show the accumulation of observed OTUs with increasing sequencing depth for bacteria (16S rRNA gene; left) and fungi (ITS; right). Each coloured curve represents an individual sample. Most curves approached a plateau, indicating that the dominant community members were generally well recovered, although some bacterial curves remained incompletely saturated, suggesting incomplete sampling of rare taxa in a subset of samples. Variation among samples likely reflects, at least in part, the field‐based sampling design, as individuals were collected from different citrus trees and therefore likely differed in age, developmental history, feeding history and microenvironmental exposure. Nevertheless, the consistent recovery of dominant taxa across samples indicates that the dataset is sufficiently robust for comparative interpretation of the major community patterns presented in this study.
**Figure S2:** 16S rRNA gene sequence alignment overview of gammaproteobacterial symbionts across stink bug host species. Multiple sequence alignment of near full‐length 16S rRNA gene sequences (V1 V9; ~1350 bp) obtained via Sanger sequencing from symbiotic Gammaproteobacteria associated with the midgut (crypt‐bearing M4 region) of four stink bug species. The upper panel shows the full‐length alignment with the position of the V3–V4 region highlighted. The lower panels display a detailed view of the corresponding aligned region (positions 283–710 bp), with nucleotide positions colour‐shaded by base identity and mismatches indicated. The V3–V4 regions obtained through Illumina MiSeq sequencing for each symbiont perfectly matched the corresponding positions (283–710 bp) within the full‐length V1–V9 sequences generated by Sanger sequencing, with no ambiguous or conflicting base calls observed. This demonstrates complete concordance between sequencing approaches and confirms the accuracy and reliability of the amplicon‐based datasets.
**Figure S3:** Near full‐length (V1–V9) 16S rRNA gene sequences (1365 bp) of gammaproteobacterial symbionts from four stink bug species with V3‐V4 region (positions 283–710 bp) highlighted in grey.
**Figure S4:** Partial *cytochrome oxidase I* (COI) nucleotide sequences of four stink bug species.
**Figure S5:** Co‐occurrence networks illustrating significant associations among bacterial and fungal operational taxonomic units (OTUs) in the gut microbiomes of four Australian stink bug species: (A) *Biprorulus bibax*, (B) *Poecilometis strigatus*, (C) *Lyramorpha rosea* and (D) *Musgraveia sulciventris* . Nodes represent individual OTUs, with bacterial taxa shown in black and fungal taxa shown in red. The size of each node is proportional to the number of connections (i.e., degree), and the thickness of each connection between two nodes (i.e., edge) is proportional to the value of Spearman's coefficients coefficient > 0.60 and *p* < 0.05. Overall, the networks were dominated by positive associations, whereas negative associations were comparatively few, and network structure differed among the four host species, indicating host‐specific patterns of microbial community organisation.
**Figure S6:** Maximum Likelihood phylogenetic tree based on partial 16S rRNA gene V3–V4 region sequences (positions 283–710; 428 bp), showing the placement of the dominant gut symbionts (GS) from four Australian stink bug species across the Enterobacteriaceae symbionts (Gammaproteobacteria) of other host species with accession numbers given. The analysis was performed in MEGA using the Kimura 2‐parameter model with gamma‐distributed rate variation and a proportion of invariant sites (K2 + G + I), identified as the best‐fit model. Nodal support values are shown as bootstrap percentages based on 1000 replicates.
**Figure S7:** Maximum Likelihood phylogenetic tree based on partial mitochondrial COI gene sequences (~615 bp), showing the placement of the four Australian stink bug species within Pentatomoidea. The analysis was conducted in MEGA using the best‐fit substitution model, GTR + G + I. Nodal support values are shown as bootstrap percentages based on 1000 replicates. Accession numbers are given after each taxon name, with the corresponding family indicated in parentheses.

**Table S1:** Metadata for stink bug (crypt‐bearing M4 midgut region) samples used in this study (sample ID, species/family, sex, coordinates, date, host plant, collector.

## Data Availability

The 16S rRNA gene and ITS amplicon sequencing datasets generated in this study are available in the NCBI Sequence Read Archive (SRA) under BioProject PRJNA1420412. Associated metadata, OTU tables, and all raw data used in this study are available on Figshare at https://doi.org/10.6084/m9.figshare.31341679. COI sequences and near full‐length 16S rRNA gene sequences (V1–V9) of the dominant gut bacterial symbionts generated in this study are available in GenBank, and the accession numbers for each sequence are provided in the manuscript. Supplementary Information contains Figure S1–S7 and Table S1.
