# Supplementary material for: Bacterial and Fungal Midgut Communities of Four Australian Citrus Stinkbug Species, With a Focus on Pantoea‐Like Symbionts and Their Fitness Effects in Biprorulus bibax
Source: Environ Microbiol. 2026 Oct 26;28(10):e70459. doi: 10.1111/1462-2920.70459 (PMC13708740; doi:10.1111/1462-2920.70459)
Supplement: Supplementary file 1 [file EMI-28-e70459-s001.pdf]

SUPPLEMENTARY MATERIAL: Figures S1-S7

TABLE OF CONTENTS

Figure S1. Rarefaction curves of bacterial and fungal communities 1
Figure S2. 16S rRNA gene sequence alignment overview of Gammaproteobacterial symbionts across four stink bug host species.... 2
Figure S3. Near full-length (V1–V9) 16S rRNA gene sequences of Gammaproteobacterial symbionts from four stink bug species 3
Figure S4. Partial cytochrome oxidase I (COI) nucleotide sequences of four stink bug species 4
Figure S5. Bacterial–fungal co-occurrence networks in Australian stink bugs 5
Figure S6. Phylogenetic placement of stink bug gut symbionts based on 16S rRNA V3–V4 sequences 6
Figure S7. Phylogenetic placement of Australian stink bugs based on partial mitochondrial COI gene sequences 7

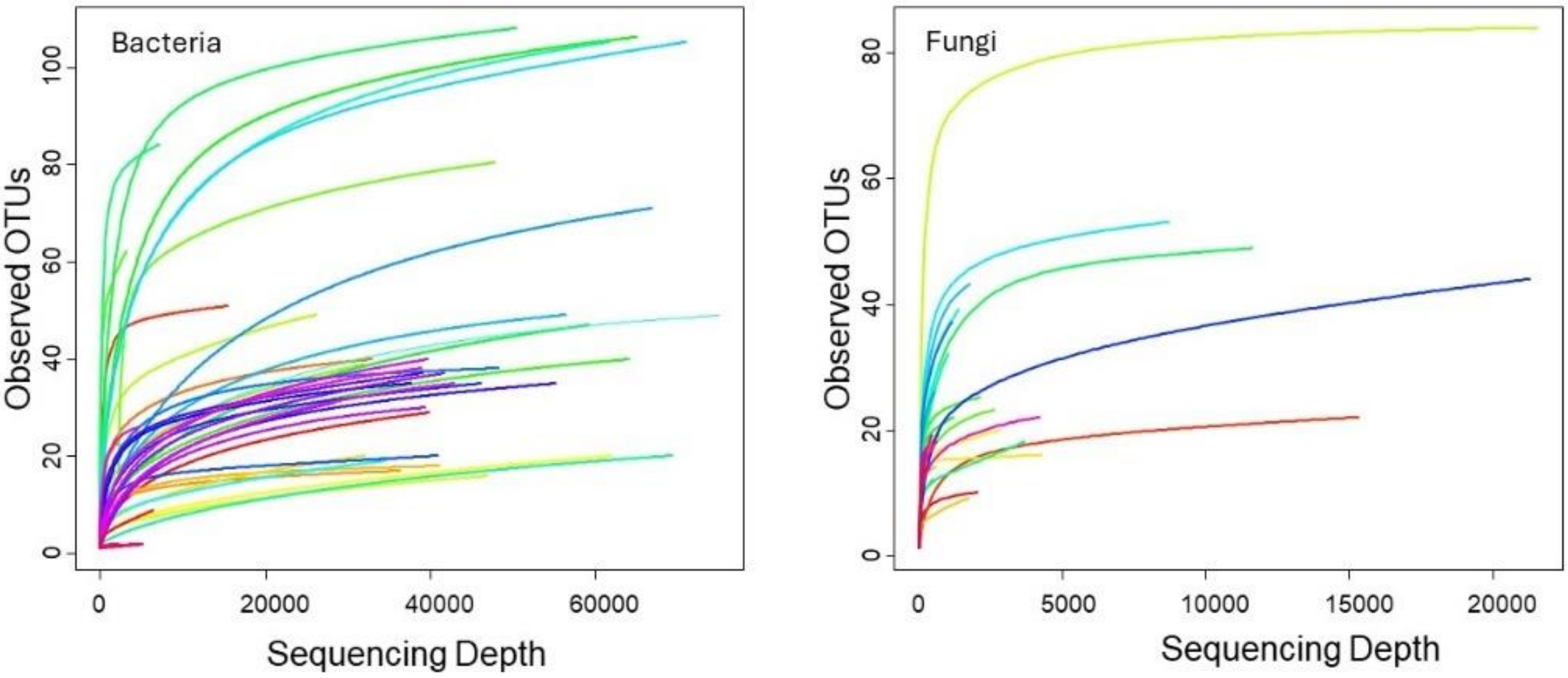

Figure S1. Rarefaction curves of bacterial and fungal communities. Rarefaction plots show the accumulation of observed OTUs with increasing sequencing depth for bacteria (16S rRNA gene; left) and fungi (ITS; right). Each coloured curve represents an individual sample. Most curves approached a plateau, indicating that the dominant community members were generally well recovered, although some bacterial curves remained incompletely saturated, suggesting incomplete sampling of rare taxa in a subset of samples. Variation among samples likely reflects, at least in part, the field-based sampling design, as individuals were collected from different citrus trees and therefore likely differed in age, developmental history, feeding history and microenvironmental exposure. Nevertheless, the consistent recovery of dominant taxa across samples indicates that the dataset is sufficiently robust for comparative interpretation of the major community patterns presented in this study.

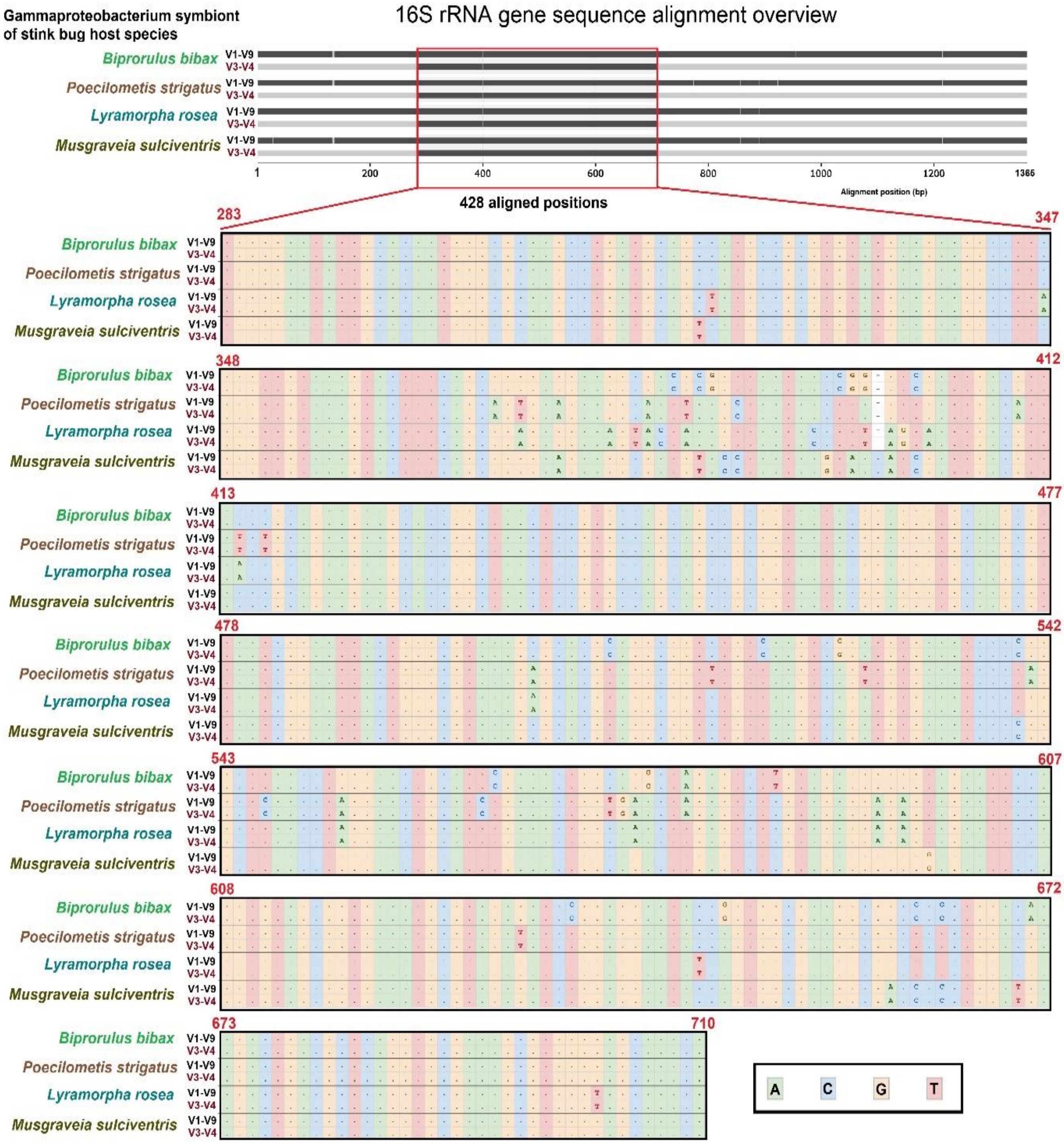

Figure S2. 16S rRNA gene sequence alignment overview of gammaproteobacterial symbionts across stink bug host species. Multiple sequence alignment of near full-length 16S rRNA gene sequences (V1–V9; ~1,350 bp) obtained via Sanger sequencing from symbiotic Gammaproteobacteria associated with the midgut (crypt-bearing M4 region) of four stink bug species. The upper panel shows the full-length alignment with the position of the V3–V4 region highlighted. The lower panels display a detailed view of the corresponding aligned region (positions 283–710 bp), with nucleotide positions colour-shaded by base identity and mismatches indicated. The V3–V4 regions obtained through Illumina MiSeq sequencing for each symbiont perfectly matched the corresponding positions (283–710 bp) within the full-length V1–V9 sequences generated by Sanger sequencing, with no ambiguous or conflicting base calls observed. This demonstrates complete concordance between sequencing approaches and confirms the accuracy and reliability of the amplicon-based datasets.

> Gammaproteobacterial symbiont of *Biprorulus bibax* (PZ019523)

GAGCTTGCTCTTCGGGTGGCGAGTGGCGGACGGGTGAGTAATGTCTGGGAAACTGCCCGATGGAGGGGGATAACTACTGGAAACGGTAGCTAATACCG
CATAACGTCGCAAGACCAAAGTGGGGGACCTTCGGGCCTCACACCATCGGATGTGCCCAGATGGGATTAGCTAGTAGGTGGGGTAACGGTTCACCTAG
GCGACGATCCCTAGCTGGTCTGAGAGGATGACCAGCCACACTGGAACTGAGACACGGTCCAGACTCCTACGGGAGGCAGCAGTGGGGAATATTGCACA
ATGGGCGCAAGCCTGATGCAGCCATGCCGCGTGTATGAAGAAGGCCTTCGGGTTGTAAAGTACTTTCAGCGGGGAGGAAGGCGACGCGGTTAATAACC
GGGTCGATTGACGTTACCCGCAGAAGAAGCACCGGCTAACTCCGTGCCAGCAGCCGCGGTAATACGGAGGGTGCAAGCGTTAATCGGAATTACTGGGC
GTAAAGCGCACGCAGGCGGTCTGTCAAGTCGGATGTGAAATCCCCGGGCTTAACCTGGGAACTGCATTCGAAACTGGCAGGCTAGAGTCTTGTAGAGG
GGGGTAGAATTCCAGGTGTAGCGGTGAAATGCGTAGAGATCCGGAGGAATACCGGTGGCGAAGGCGGCCCCCTGGACAAAGACTGACGCTCAGGTGC
GAAAGCGTGGGGAGCAAACAGGATTAGATACCCTGGTAGTCCACGCCGTAAACGATGTCGACTTGGAGGCTGTTCCCTTGAGGAGTAGCTTCCGGAGC
TAACGCGTTAAGTCGACCGCCTGGGGAGTACGGCCGCAAGGTTAAAACTCAAATGAATTGACGGGGGCCCGCACAAGCGGTGGAGCATGTGGTTTAA
TTTCGATGCAACGCGAAGAACCTTACCTACTCTTGACATCCAGGGCACTTGCCAGAGATGGCTTGGTCCTTCGGGAACTCTGAGACAGGTGCTGCATGG
CTGTCGTCAGCTCGTGTTGTGAAATGTTGGGTTAAGTCCCGCAACGAGCGCAACCCTTATCCTTTGTTGCCAGCGATTCGGTCGGGAACTCAAAGGAGA
CTGCCGGTGATAAACCGGAGGAAGGTGGGGATGACGTCAAGTCATCATGGCCCTTACGAGTAGGGCTACACACGTGCTACAATGGCGCATACAAAGAG
AAGCGACCTCGCGAGAGCAAGCGGACCTTATAAAGTGCGTCGTAGTCCGGATCGGAGTCTGCAACTCGACTCCGTGAAGTCGGAATCGCTAGTAATCG
TGGATCAGAATGCCACGGTGAATACGTTCCCGGGCCTTGTACACACCGCCCGTCACACCATGGGAGTGGGTTGCAAAAGAAGT

> Gammaproteobacterial symbiont of *Poecilometis strigatus* (PZ019524)

GAGCTTGCTCTTTGGATGGCGAGTGGCGAACGGGTGAGTAATATCTGGGAAACTACCCAATGGAGGGGGATAACTATTGGAAACGATAGCTAATACCG
CATAATGTCGTAAGACCAAAGTGGGGGACCTTATGGCCTCATACCATTGGATGTGCCCAGATGAGATTAGCTAGTAGGTGAGGTAATGGCTCACCTAGG
CTACGATCTCTAGCTGGTCTGAGAGGATGACCAGCCACACTGGAACTGAGACACGGTCCAGACTCCTACGGGAGGCAGCAGTGGGGAATATTGCACAA
TGGGCGCAAGCCTGATGCAGCCATGCCGCGTGTATGAAGAAGGCCTTCGGGTTGTAAAGTACTTTCAGCAGTGAAGAAGGCAATTAAGCTAATAACTT
AGTTGATTGACATTATCTGCAGAAGAAGCACCGGCTAACTCCGTGCCAGCAGCCGCGGTAATACGGAGGGTGCAAGCGTTAATCGGAATTACTGGGCG
TAAAGAGCACGTAGGCGGTTTGTTAAGTCAGTTGTGAAATCCCTAGGCTCAACCTAGGAACTGCATCTGAAACTGGTGAACTAGAGTCTCGTAGAGGA
GAGTAGAATTCCAGGTGTAGCGGTGAAATGCGTAGATATCTGGAGGAATACCAGTGGCGAAGGCGGCTCTCTGGACGAAGACTGACGCTCAGGTGCGA
AAGCGTGGGGAGCAAACAGGATTAGATACCCTGGTAGTCCATGCTGTAAACGATGTCGACTTGGAGGTTGTATTATATAATATGGCTTCCGTAGCTAAC
GCGTTAAGTCGACCGCCTGGGGAGTACGACCGCAAGGTTAAAACTCAAATGAATTGACGGGTGCCCGCACAAGCGGTGGAGCATGTGGTTTAATTCGA
TGCAACGCGAAGAACCTTACCTGGCCTTGAATCCAGAGAATTTTTTAGAAATAGAAAAGTGCCTTGCGGAACTCTGAGACAGGTGCTGCATGGCTGTCG
TCAGCTCGTGTTGTGAAATGTTGGGTTAAGTCCCGCAACGAGCGTAACCCTTATCCTTTGTTGCCAGCGGTTCGGCCGGGAACTCAAAGGAGACTGCCG
GTGATAAACCGGAGGAAGGTGAGGATGACGTCAAGTCATCATGGCCCTTACGGCCAGGGCTACACACGTGCTACAATGGTATATACAAAGAGAAGCAA
CCTTGTAAAAGTCAGCCAATCTCACAAAATATATCGTAGTTCGGATTGGAGTCTGCAACTCGACTCCATGAAGTCGGAATCGCTAGTAATCGTAGATCA
GAATGCTACGGTGAATACGTTCTCGGGCCTTGTACACACCGCCCGTCACACCATGGAAGTGGGTTGCAAAAGAAGT

> Gammaproteobacterial symbiont of *Lyramorpha rosea* (PZ019525)

AAGCTTGCTTCTTAGGTGACGAGTGGCGGACGGGTGAGTAATGTCTGGGGATCTGCCTTATGGAGGGGGATAACTACTGGAAACGGTAGCTAATACCG
CATAATGTCAAAAGACCAAAGTGGGGGACCTTATAAAAGGCCTCACACCATTAGATGAACCCAGATGGGATTAGCTAGTAGGTGAGGTAAAGGCTCAC
CTAGGCCACGATCCCTAGCTGGTCTGAGAGGATGGCCAGCCACACTGGAACTGAGACACGGTCCAGACTCCTACGGGAGGCAGCAGTGGGGAATATTG
CACAATGGGCGCAAGCCTGATGCAGCTATGCCGCGTGTATGAAGAAGGCCTTAGGGTTGTAAAGTACTTTCAGCGGAGAGGAAAGTACTAAAGTTAAT
ACCTTTAGTAATTGACGTTAACCGCAGAAGAAGCACCGGCTAACTCCGTGCCAGCAGCCGCGGTAATACGGAGGGTGCAAGCGTTAATCGGAATTACT
GGGCGTAAAGAGCACGTAGGCGGTCTGTTAAGTCAGATGTGAAATCCCTGGGCTTAACCTAGGAACTGCATTTGAAACTGGCAAACTTGAGTCTCGTAG
AGGAGAGTAGAATTCCAGGTGTAGCGGTGAAATGCGTAGAGATCTGGAGGAATATCAGTGGCGAAGGCGGCTCTCTGGACGAAGACTGACGCTCAGGT
GCGAAAGCGTGGGTAGCAAACAGGATTAGATACCCTGGTAGTCCATGCCGTAAACGATGTCGACTTGGAGGTTGTGCCCTTGAGGTGTGGCTTCCGGA
GCTAACGCATTAAGTCGACCGCCTGGGGAGTACGGCCGCAAGGTTAAAACTCAAATGAATTGACGGGGGTCCGCACAAGCGGTGGAGCATGTGGTTTA
ATTCGATGCAACACGAAGAACCTTACCTACTCTTGACATCCAGAGAACGAACTAGAGATAGTTTGATGCCTTAGGGAACTCTGAGACAGGTGCTGCATG
GCTGTCGTCAGCTCGTGTTGTGAAATGTTGGGTTAAGTCCCGCAACGAGCGCAACCCTTATCCTTTGTTGCCAGCGACTAGGTCGGGAACTTAAAGGTG
ACTGCCGGTGATAAACCGGAGGAAGGTGGGGATGACGTCAAGTCATCATGGCCCTTACGAGTAGGGCTACACACGTGCTACAATGGCTCATACAAAGA
GATGCAACCTCGCGAGAGTAAGCGGATCTCATTAAAGTGAGTCGTAGTCCGGATTGGAGTCTGCAACTCGACTCCATGAAGTAGGAATCGCTAGTAATC
GTGGATCAGAATGCCACGGTGAATACGTTCCCGGGCCTTGTACACACCGCCCGTCACACCATGGGAGTGGGTTGCAAAAGAAGT

> Gammaproteobacterial symbiont of *Musgraveia sulciventris* (PZ019526)

GCTTTTGCTCCTTAGGTGACGAGTGGGGAAGGGTGAGTAATGTCTGGGGATCTGCCCAATGGAGGGGGATAACCACTGGAAACGGTGGCTAATACCGC
ATAATGTCGTAAGACCAAAGTGGGGGACCTTTAGGCCTCACGCCATCGGATGAACCCAGATGGGATTAGCTAGTAGGTGAGGTAATGGCTCACCTAGG
CGACGATCCCTAGCTGGTCTGAGAGGATGACCAGCCACACTGGAACTGAGACACGGTCCAGACTCCTACGGGAGGCAGCAGTGGGGAATATTGCACAA
TGGGCGCAAGCCTGATGCAGTCATGCCGCGTGTATGAAGAAGGCCTTCGGGTTGTAAAGTACTTTCAGCGGGGAAGAAGGCGATGTACCTAATAAGTA
ACATCGATTGACGTTACCCGCAGAAGAAGCACCGGCTAACTCCGTGCCAGCAGCCGCGGTAATACGGAGGGTGCAAGCGTTAATCGGAATTACTGGGC
GTAAAGCGCACGTAGGCGGTCTGTTAAGTCAGATGTGAAATCCCCGGGCTTAACCTGGGAACTGCATTTGAAACTGGCAGACTTGAGTCTCGTAGAGG
GGGGGAGAATTCCAGGTGTAGCGGTGAAATGCGTAGAGATCTGGAGGAATACCAGTGGCGAAGGCGACCCCCTGGATGAAGACTGACGCTCAGGTGC
GAAAGCGTGGGGAGCAAACAGGATTAGATACCCTGGTAGTCCACGCTGTAAACGATGTTGACTTGGAAGTTGTATCCTTGAGATGTGGCTTCCGGAGCT
AACGCGTTAAGTCAACCGCCTGGGGAGTACGGCCGCAAGGTTAAAACTCAAATGAATTGACGGGGGCCCGCACAAGCGGTGGAGCATGTGGTTTAATT
CGATGCAACGCGAAGAACCTTACCTATTCTTGACATCCAGAGAACTCTCTAGAGATGGATTGGTGCCTTCGGGAACTCTGAGACAGGTGCTGCATGGCT
GTCGTCAGCTCGTGTTGTGAAATGTTGGGTTAAGTCCCGCAACGAGCGCAACCCTTGTCTTTTGTTGCCAGCGATTCGGTCGGGAACTCAAAAGAGACT
GCCGGTGATAAACCGGAGGAAGATGGGGATGACGTCAAGTCATCATGGCCCTTACGAGTAGGGCTACACACGTGCTACAATGGTGCATACAAAGAGAA
GCGACCTCGTAAGAGCAAGCAGACCTCATAAAGTGCGTCGTAGTTCGGATTGGAGTCTGCAACTCGACTCCATGAAGTCGGAATCGCTAGTAATCGTGG
ATCAGAATGCCACGGTGAATACGTTCCCGGGCCTTGTACACACCGCCCGTCACACCATGGGAGTGGGTTGCAAAAGAAGT

Figure S3. Near full-length (V1–V9) 16S rRNA gene sequences (1,365 bp) of gammaproteobacterial symbionts from four stink bug species with V3-V4 region (positions 283–710 bp) highlighted in grey.

> COI of *Biprorulus bibax* - 615 bp (PZ019527)

GGATCAGCCATAAGAGTAATTATCCGAATTGAATTAAGACAACCAGGAAGATTCATTGGCAATGATCAAATCTATAATGTAATCGT
CACAGCACATGCATTCGTAATAATTTTCTTTATAGTAATACCAATCATGATTGGAGGATTCGGTAATTGATTAGTACCATTAATAAT
CGGAGCTCCTGATATAGCATTCCCACGATTAAATAATATAAGATTCTGATTATTACCCCCCTCTATCACATTACTTATAGTAAGTAG
ACTAGCAGAGACAGGAGTAGGAACTGGTTGAACAGTATATCCTCCTTTATCAAGAAATACAGCTCATGCTGGAGCTCCAGTAGATT
TAGCAATTTTCTCACTACACCTAGCAGGTGTATCATCTATTCTAGGTGCAGTAAATTTTATCTCAACAATTATCAATATACGACCAGA
AGGAATAACACCTGAACGAATCCCTCTATTCGTATGATCAGTAAGAATTACCGCATTATTATTACTGCTATCTTTACCAGTGCTAGC
AGGTGCTATCACTATACTATTAACAGATCGTAATTTTAATACCTCATTCTTTGACCCATCAGGAGGAGGGGACCCAATTTTGTATCA
ACACTTA

> COI of *Poecilometis strigatus* - 615 bp (PZ019530)

GGATCAGCTATAAGAGTTATTATTCGAATTGAATTAGGTCAACCTGGAAGATTTATTGGAGATGATCAAATTTATAATGTCATAGTG
ACAGCTCACGCATTTATTATAATTTTTTTTATAGTAATGCCAATTATAATTGGAGGATTTGGAAATTGACTAGTACCTTTAATAATTG
GAGCTCCTGATATAGCATTCCCTCGAATGAATAATATAAGATTTTGATTATTACCTCCATCACTAACCTTATTATTAATAAGAAGATT
AACTGAATCAGGAGCAGGTACAGGATGAACGGTTTATCCACCCTTATCAAGTAATATTTCCCATACAGGAGCTTCAGTTGATCTAGC
TATTTTTTCACTTCATTTAGCAGGAATTTCATCAATTTTAGGAGCAGTAAACTTCATTTCAACAATTATTAATATACGACCTGCTGGC
ATAACCCCTGAACGAATCCCTCTCTTTGTATGATCAGTAGTAATTACAGCTTTATTACTCTTATTATCTTTACCTGTTTTAGCAGGAG
CTATTACTATACTTTTAACAGATCGTAATTTTAATACTTCATTTTTTGATCCCTCTGGAGGAGGTGACCCTATTTTATATCAACATTTA

> COI of *Lyramorpha rosea* - 615 bp (PZ019533)

GGAACCGCAATAAGATGAATTATCCGAATCGAATTAGGACAACCAGGAACATTTATTGGAGATGATCAAATTTATAATGTAATTGT
AACTGCCCACGCATTCGTTATAATTTTCTTTATAGTAATACCCATCATAATTGGAGGGTTCGGAAATTGACTAGTACCATTAATAATT
GGAGCCCCCGATATGGCATTTCCTCGAATAAATAATATAAGATTCTGACTATTACCCCCTTCATTAACACTGCTATTAATAAGAAGA
ATAGTAGAAGCAGGAGTGGGGACAGGATGAACAGTATACCCGCCACTATCGAGAAACATAGCCCATAGAGGAGCATCAGTAGATA
TAGCCATCTTCTCACTTCACCTAGCTGGGATTTCCTCAATTTTAGGAGCAGTGAACTTTATTTCAACAATTATAAACATACGACCCGC
AGGAATAACACCTGAACGAATCCCTCTATTTGTATGGTCAGTAGGAATTACAGCGATATTACTTTTATTGTCACTCCCTGTATTAGC
AGGGGCTATCACTATATTATTAACCGATCGAAATTTTAATACCTCACTCTTTGAGCCCTCAGGAGGGGGTGACCCAATTTTATATCA
ACACTTA

> COI of *Musgraveia sulciventris* - 601 bp (PZ274252)

ATGAATTATCCGAGTAGAACTAGGGCAACCCGGAACATTTATTGGAGATGATCAAATTTACAATGTAGTAGTAACAGCTCATGCAT
TTGTAATAATCTTCTTTATAGTAATACCAGTAATAATCGGAGGATTTGGCAACTGACTAGTACCTCTAATAATTGGGGCCCCAGATA
TAGCATTCCCACGGATAAATAATATAAGATTCTGATTATTACCCCCCTCTCTAACTCTACTAATAATAAGAAGAATAGTAGAAACCG
GGGCAGGGACTGGGTGAACAGTGTATCCCCCTCTATCAAGTAACCTGTCACACAGAGGAGCATCTGTAGACTTGGCTATTTTTTCCC
TGCACCTAGCAGGAGTGTCCTCTATCCTAGGAGCTGTGAATTTTATTTCCACCATTGTAAACATGCGACCCGCTGGGATAACACCCG
AACGGGTGCCCCTATTTGTCTGATCAGTAGGAATTACAGCGTTACTATTACTATTATCCCTGCCAGTATTGGCCGGGGCTATCACAA
TACTATTAACTGATCGAAATTTTAACACCTCATTCTTTGATCCCTCAGGGGGAGGAGATCCAATTTTATATCAACACTTA

Figure S4. Partial *cytochrome oxidase I* (COI) nucleotide sequences of four stink bug species.

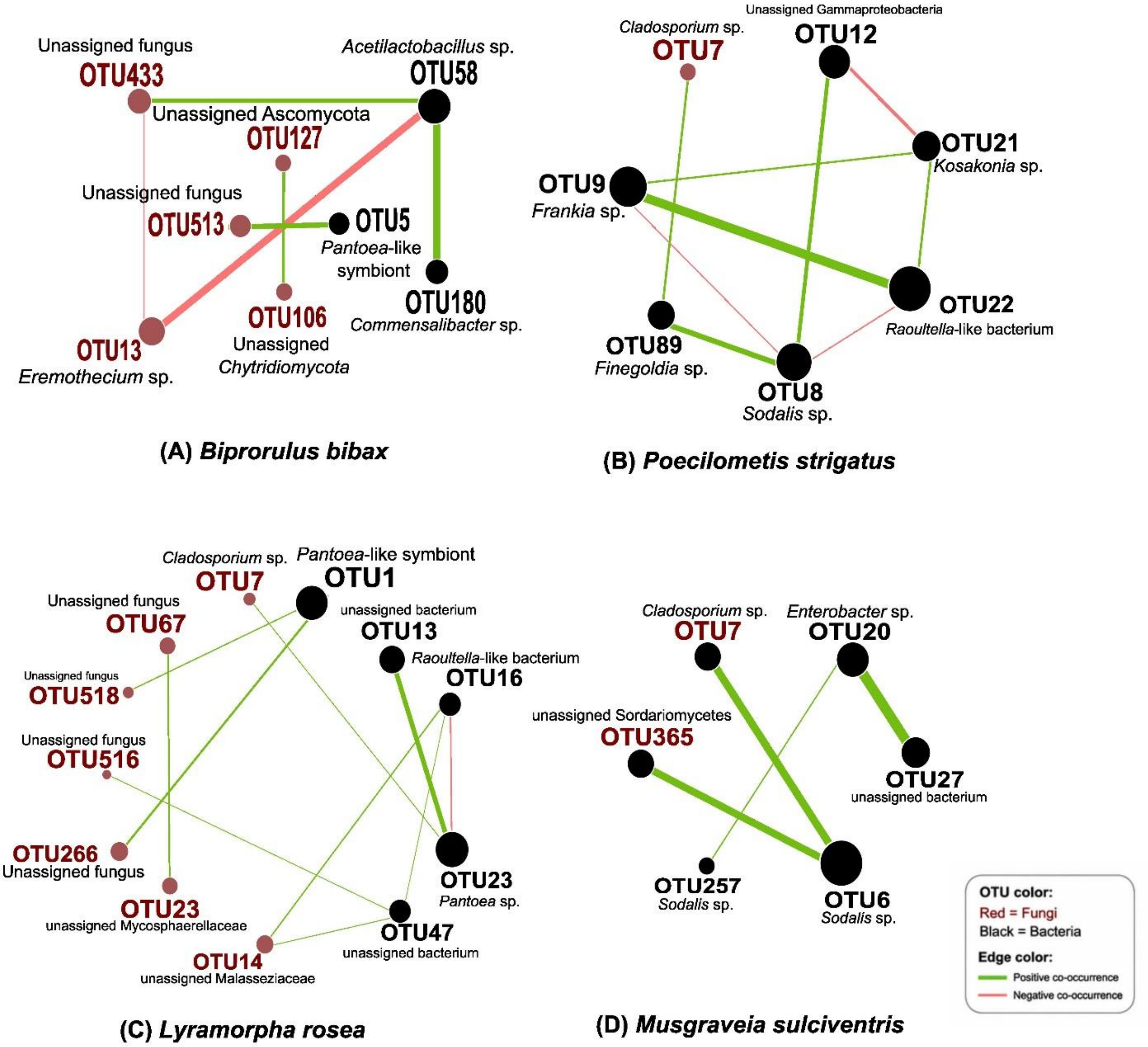

Fig. S5. Co-occurrence networks illustrating significant associations among bacterial and fungal operational taxonomic units (OTUs) in the gut microbiomes of four Australian stink bug species: **(A)** *Biprorulus bibax*, **(B)** *Poecilometis strigatus*, **(C)** *Lyramorpha rosea*, and **(D)** *Musgraveia sulciventris*. Nodes represent individual OTUs, with bacterial taxa shown in black and fungal taxa shown in red. The size of each node is proportional to the number of connections (i.e. degree), and the thickness of each connection between two nodes (i.e. edge) is proportional to the value of Spearman's coefficients coefficient >0.60 and $p < 0.05$. Overall, the networks were dominated by positive associations, whereas negative associations were comparatively few, and network structure differed among the four host species, indicating host-specific patterns of microbial community organisation.

Host-specific co-occurrence network analysis revealed distinct patterns of bacterial–fungal associations across the four stink bug species (Fig. S2). In *B. bibax* **(A)**, the network was relatively simple, with the dominant *Pantoea*-like symbiont (OTU5) showing weak positive association with a low-abundance fungal OTU513. In *P. strigatus* **(B)**, the network was more structured, with strong positive associations among bacterial taxa, particularly between *Frankia* sp. (OTU9) and a *Raoultella*-like bacterium (OTU22). The network for *L. rosea* **(C)** was the most complex, showing numerous interactions among both bacterial and fungal OTUs. The dominant *Pantoea*-like symbiont (OTU1) was connected to unassigned fungal OTU266 and OTU518. In *M. sulciventris* **(D)**, the network was simpler and more modular, dominated mainly by strong positive associations among bacterial taxa, particularly *Sodalis* spp. (OTU6 and OTU257) and *Enterobacter* sp. (OTU20) and *Cladosporium* sp. (OTU7), with comparatively limited fungal interactions overall.

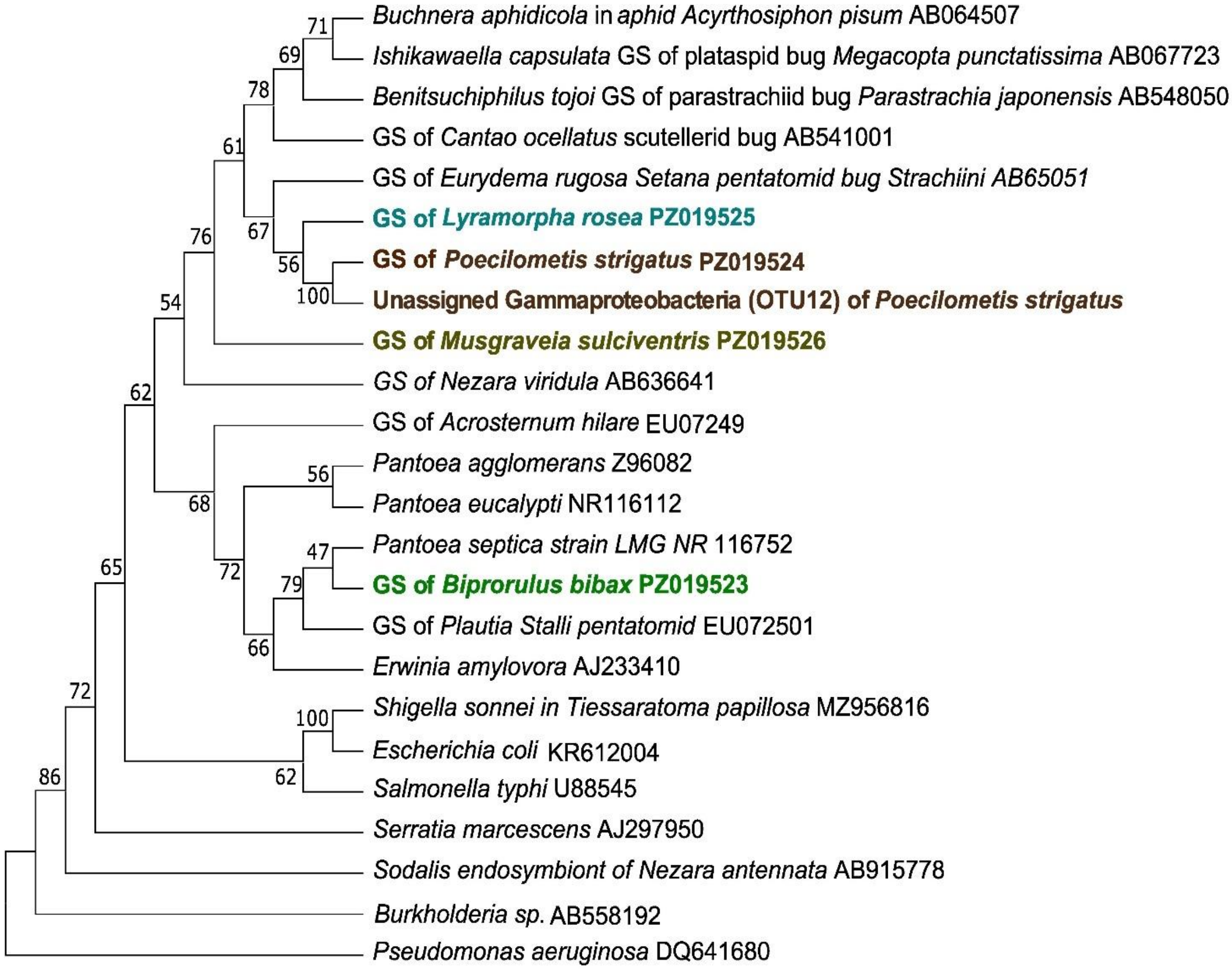

Fig. S6. Maximum Likelihood phylogenetic tree based on partial 16S rRNA gene V3–V4 region sequences (positions 283–710; 428 bp), showing the placement of the dominant gut symbionts (GS) from four Australian stink bug species across the Enterobacteriaceae symbionts (Gammaproteobacteria) of other host species with accession numbers given. The analysis was performed in MEGA using the Kimura 2-parameter model with gamma-distributed rate variation and a proportion of invariant sites (K2+G+I), identified as the best-fit model. Nodal support values are shown as bootstrap percentages based on 1,000 replicates.

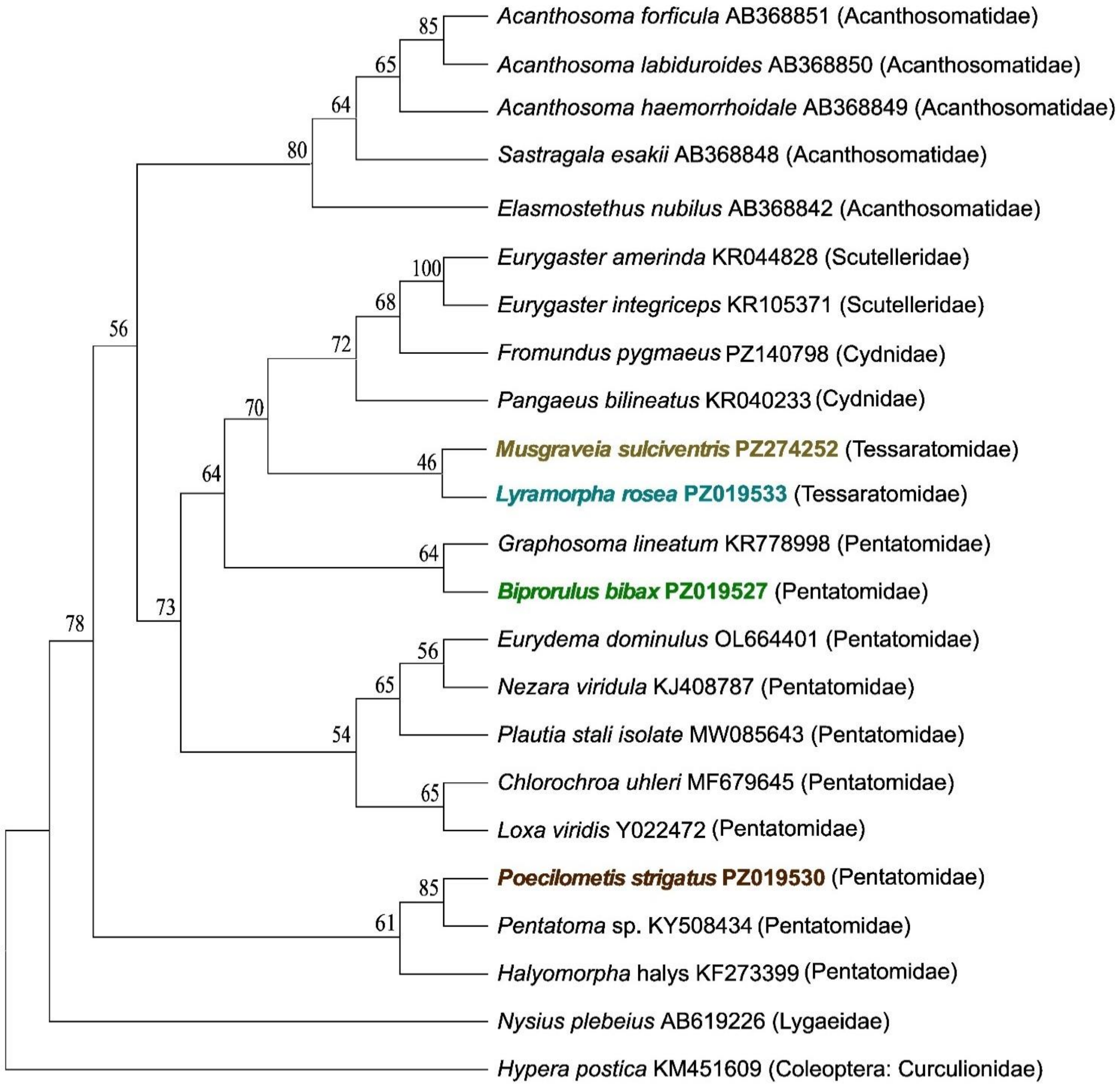

Fig. S7. Maximum Likelihood phylogenetic tree based on partial mitochondrial COI gene sequences (~615 bp), showing the placement of the four Australian stink bug species within Pentatomoidea. The analysis was conducted in MEGA using the best-fit substitution model, GTR+G+I. Nodal support values are shown as bootstrap percentages based on 1,000 replicates. Accession numbers are given after each taxon name, with the corresponding family indicated in parentheses.
