# Supplementary material for: Bacterial and Fungal Midgut Communities of Four Australian Citrus Stinkbug Species, With a Focus on Pantoea‐Like Symbionts and Their Fitness Effects in Biprorulus bibax
Source: Environ Microbiol. 2026 Oct 26;28(10):e70459. doi: 10.1111/1462-2920.70459 (PMC13708740; doi:10.1111/1462-2920.70459)
Supplement: Supplementary file 2 [file EMI-28-e70459-s002.pdf]

SUPPLEMENTARY MATERIAL

Table S1. Metadata for stink bug (crypt-bearing M4 midgut region) samples used in this study (sample ID, species/family, sex, coordinates, date, host plant, collector.

Sample	Species name	Family	Extracted tissue	Sex	Coordinates	Date	Host plant	Col.
**SCB1**	*Biprorulus bibax*	Pentatomidae	M4 region (crypts)	Female	33°36'S 150°44'E	Jun-21	Mandarin	A. Katlav
**SCB2**	*Biprorulus bibax*	Pentatomidae	M4 region (crypts)	Female	33°36'S 150°44'E	Jun-21	Mandarin	A. Katlav
**SCB3**	*Biprorulus bibax*	Pentatomidae	M4 region (crypts)	Female	33°36'S 150°44'E	Jun-21	Mandarin	A. Katlav
**SCB4**	*Biprorulus bibax*	Pentatomidae	M4 region (crypts)	Female	33°36'S 150°44'E	Jun-21	Lemon	A. Katlav
**SCB5**	*Biprorulus bibax*	Pentatomidae	M4 region (crypts)	Male	33°36'S 150°44'E	Jun-21	Lemon	A. Katlav
**SCB6**	*Biprorulus bibax*	Pentatomidae	M4 region (crypts)	Female	33°36'S 150°44'E	Jun-21	Mandarin	A. Katlav
**SCB7**	*Biprorulus bibax*	Pentatomidae	M4 region (crypts)	Male	33°36'S 150°44'E	Jun-21	Mandarin	A. Katlav
**SCB8**	*Biprorulus bibax*	Pentatomidae	M4 region (crypts)	Female	33°36'S 150°44'E	Jun-21	Mandarin	A. Katlav
**SCB9**	*Biprorulus bibax*	Pentatomidae	M4 region (crypts)	Male	33°36'S 150°44'E	Jun-21	Orange	A. Katlav
**SCB10**	*Biprorulus bibax*	Pentatomidae	M4 region (crypts)	Male	33°36'S 150°44'E	Jun-21	Orange	A. Katlav
**GTB1**	*Poecilometis strigatus*	Pentatomidae	M4 region (crypts)	Female	33°36'S 150°44'E	Jun-21	Mandarin	A. Katlav
**GTB2**	*Poecilometis strigatus*	Pentatomidae	M4 region (crypts)	Male	33°36'S 150°44'E	Jun-21	Mandarin	A. Katlav
**GTB3**	*Poecilometis strigatus*	Pentatomidae	M4 region (crypts)	Female	33°36'S 150°44'E	Jun-21	Mandarin	A. Katlav
**GTB4**	*Poecilometis strigatus*	Pentatomidae	M4 region (crypts)	Female	33°36'S 150°44'E	Jun-21	Mandarin	A. Katlav
**GTB5**	*Poecilometis strigatus*	Pentatomidae	M4 region (crypts)	Female	33°36'S 150°44'E	Jun-21	Mandarin	A. Katlav
**LB1**	*Lyramorpha rosea*	Tessaratomidae	M4 region (crypts)	Female	33°36'S 150°44'E	Jun-21	Mandarin	A. Katlav
**LB2**	*Lyramorpha rosea*	Tessaratomidae	M4 region (crypts)	Male	33°36'S 150°44'E	Jun-21	Mandarin	A. Katlav
**LB3**	*Lyramorpha rosea*	Tessaratomidae	M4 region (crypts)	Male	33°36'S 150°44'E	Jun-21	Mandarin	A. Katlav
**LB4**	*Lyramorpha rosea*	Tessaratomidae	M4 region (crypts)	Female	33°36'S 150°44'E	Jun-21	Grapefruit	A. Katlav
**LB5**	*Lyramorpha rosea*	Tessaratomidae	M4 region (crypts)	Female	33°36'S 150°44'E	Jun-21	Mandarin	A. Katlav
**LB6**	*Lyramorpha rosea*	Tessaratomidae	M4 region (crypts)	Female	33°36'S 150°44'E	Jun-21	Grapefruit	A. Katlav
**LB7**	*Lyramorpha rosea*	Tessaratomidae	M4 region (crypts)	Female	33°36'S 150°44'E	Jun-21	Grapefruit	A. Katlav
**LB8**	*Lyramorpha rosea*	Tessaratomidae	M4 region (crypts)	Female	33°36'S 150°44'E	Jun-21	Mandarin	A. Katlav
**LB9**	*Lyramorpha rosea*	Tessaratomidae	M4 region (crypts)	Female	33°36'S 150°44'E	Jun-21	Mandarin	A. Katlav
**BOB1**	*Musgraveia sulciventris*	Tessaratomidae	M4 region (crypts)	Female	33°36'S 150°44'E	Jun-21	Lemon	A. Katlav
**BOB2**	*Musgraveia sulciventris*	Tessaratomidae	M4 region (crypts)	Female	33°36'S 150°44'E	Jun-21	Orange	A. Katlav
**BOB3**	*Musgraveia sulciventris*	Tessaratomidae	M4 region (crypts)	Female	33°36'S 150°44'E	Jun-21	Orange	A. Katlav
**BOB4**	*Musgraveia sulciventris*	Tessaratomidae	M4 region (crypts)	Female	33°36'S 150°44'E	Jun-21	Orange	A. Katlav
**BOB5**	*Musgraveia sulciventris*	Tessaratomidae	M4 region (crypts)	Female	33°36'S 150°44'E	Jun-21	Orange	A. Katlav
**BOB6**	*Musgraveia sulciventris*	Tessaratomidae	M4 region (crypts)	Male	33°36'S 150°44'E	Jun-21	Orange	A. Katlav
**BOB7**	*Musgraveia sulciventris*	Tessaratomidae	M4 region (crypts)	Male	33°36'S 150°44'E	Jun-21	Lemon	A. Katlav
**BOB8**	*Musgraveia sulciventris*	Tessaratomidae	M4 region (crypts)	Female	33°36'S 150°44'E	Jun-21	Lemon	A. Katlav
